# Supplementary material for: Exploring menstrual products: A systematic review and meta-analysis of reusable menstrual pads for public health internationally
Source: PLoS One. 2021 Sep 24;16(9):e0257610. doi: 10.1371/journal.pone.0257610 (PMC8462722; doi:10.1371/journal.pone.0257610)
Supplement: S1 File — (PDF) [file pone.0257610.s002.pdf]

Supplement:

Exploring Menstrual Products: a Systematic Review and Meta-analysis of Reusable Menstrual Pads for Public Health Internationally

Contents

1. Supplemental methods 2
Additional information on the search 2
Additional meta-analysis methods for quantitative studies 3
Methods qualitative analysis 3
Methods visibility and availability 3
Methods for comparison of costs and waste 3
Changes with protocol 3
Table S1. Adaptation of quality assessment form for observational studies 4

2. Additional Results 5
Table S2. Search results databases from inception to 1 November 2020 5
Table S3. Quality assessment of the studies involved (studies with quantitative information) 5
Table S4. Manufacturers, city, country, and websites for reusable pads used in included studies 7
Table S5. Studies with other types of information contributing to the Reusable Menstrual Pad review (alphabetical order) 7

Section 2.1. Qualitative studies 8
Table S6. CASP-based appraisal of qualitative studies* 8
Table S7. Characteristics of qualitative papers 9
Table S8. Themes in qualitative studies 12

Section 2.2 Acceptability 18
Table S9. Reasons for not using reusable menstrual pads in surveys 18
Figure S1: Reported use of reusable pads in surveys in Ugandan schools, 5 studies, 2014-2019 18
Figure S2. Use of reusable pads by follow up time, 19 studies, 2014-2019 19
Figure S3. Uptake of reusable menstrual pads in schools by brand, Africa, 2014-2018 19

Section 2.3 RMP and education 20
Table S10. Effect of reusable menstrual pads on school attendance 20

Section 2.4 RMP development 21

Section 2.5 Visibility, availability, costs and waste 22
Table S11. Studies with information on visibility or a potential interest in reusable menstrual pads 22
Table S12. Menstrual items reported in education material for girls 23
Figure S4. Menstrual items mentioned in education material on menarche, from 80 websites 25
Table S13. Brands of reusable menstrual pads, price and lifespan estimate (where available) 26
Costs and availability: Summary of findings from websites 28
Table S14. Calculations of costs per sanitary item over 2.5 and 5 years 29
Figure S5. Estimation of costs of different products used for menstruation over 2.5 and 5 years compared with RMPs with a life span of 2.5 years in LMIC and 5 years in HIC 30
Figure S6. Estimation of waste of different products used for menstruation over 2.5 and 5 years 30

References 31

Abbreviations

CASP	Critical Appraisal Skills Program
CI	Confidence interval
HIC	High income country
LMIC	Low- and middle-income country
MHM	Menstrual hygiene management
RMP	Reusable menstrual pad
US$	United States dollars

1. Supplemental methods

Additional information on the search

Search strategy: Except for PubMed, we introduced the keywords in the databases in groups (e.g., cloth* OR towel* OR pad OR suppl*) and combined searches at a second stage. In PubMed, menstrual, menses, and menstruation were used as Mesh terms and "all fields" terms. To check for completeness, we screened several other databases (See Table S2) for eligible items which were not yet included in the list resulting from the searched databases. Reference lists of relevant studies, websites of pertinent professional bodies (e.g. FDA), non-governmental organizations and 'grey literature' (e.g., reports or conference abstracts) were searched, as well as records recommended by contacting experts, to encompass a broad range of available literature. Using pre-tested forms, we extracted data on baseline characteristics and outcomes. Records from the different searches were merged, and duplicates removed; a first screening was conducted to assess potential eligibility whereby irrelevant records were removed, based on the material available (abstract or whole text). Full text articles were obtained as much as possible for the remaining articles, and these were further screened for eligibility.

Inclusion and Exclusion criteria

**Inclusion criteria**	**Exclusion criteria**
• Any study with information on use, safety, effectiveness, efficacy, or acceptability of externally applied commercially available reusable sanitary items (Cohorts, trials or cross-sectional studies; qualitative, quantitative or mixed method studies)<br>• Menstrual hygiene does not need to be the main topic, e.g. studies that are not completely about menstrual hygiene, but present useful information can still be included<br>• English language material. For articles retrieved in other languages, an attempt may be done to verify content for inclusion if a person can be identified who masters that language	• No information on externally applied commercially available reusable sanitary items (e.g. the material only covers menstrual cup or vaginal sponges or tampons, or disposable pads)<br>• Animal studies<br>• Studies where substances other than menstrual blood is collected (e.g. urine or faecal incontinence)<br>• Studies where a menstrual item is used to collect menstrual blood, or cervical or vaginal secretions, without information on effectiveness, acceptability or safety of the reusable item (as reported by the study participants)

Study characteristics included study design, number of participants, age, location, country, time-period of study, brand of reusable item involved, and type of menstrual item used before study, in addition to inclusion and exclusion criteria, and follow up time where applicable. If the year of study was not reported in an eligible study, we assumed it to be two years before the publication date. For studies with outcomes after e.g. cycle 1, cycle 2 and cycle 3, the result for the last cycle was taken. For some studies, additional information from the authors could be obtained [1]. The Picots-framework is presented below.

PICOTS Framework

**Components**	
Population	Menstruating women (including adolescent girls and menstruating transgender persons)
Intervention	Reusable sanitary item: "Items that are externally worn to absorb menstrual flow, which can be washed, dried and re-used" [2]. To differentiate from cloths made at home from sari's, blankets etc, the items must be commercially available and externally applied (as opposed to vaginal for menstrual cup and sponges).
Control	Other methods for menstrual hygiene management
Outcomes	Efficacy/effectiveness<br>1. Prevention of leakage (leaking of blood through garment)<br>Acceptability<br>1. Uptake of use<br>2. Ease of use: changing (number of changes per day, ease of changing), washing, drying and carrying (clean or used)<br>3. Comfort when wearing and odour prevention<br>4. Ability to wash and dry<br>Safety<br>1. Serious adverse events<br>2. Effect on vagina and vaginal flora<br>3. Effect on reproductive or urinary tract<br>4. Effect on the vulval skin or the groin<br>5. Safety when sanitary conditions are not optimal (lack of water, e.g. refugee camps)<br>6. Other<br>Impact:<br>• Psychological<br>• Education<br>Visibility:<br>• Information available on reusable items in education material for girls approaching menarche (separate searches)<br>Availability, costs and waste (separate searches):<br>• Products on the market<br>• Products available online/in store<br>• Materials used for products and degradability /compostability
Timing	No restriction
Setting	Any trial, quasi-experimental study, survey or other type of study reporting on experiences with reusable non-insertable material for menstruation. No restriction for language will be made, but the search will be in English.

Additional meta-analysis methods for quantitative studies

When using meta-analysis, the extent of heterogeneity was measured using the I^2 statistic [3], which is a measure of the proportion of total variability explained by heterogeneity rather than chance expressed as a percentage, with 0–40% representing no or little heterogeneity, 30–60% moderate heterogeneity, 50–90% substantial heterogeneity, and 75–100% considerable heterogeneity [4]. We examined the following sources of heterogeneity if sufficient data were available using subgroup analysis: setting of the study (high-income [HIC] versus LMIC), study population (adult women versus adolescent girls 10-19 years), water availability in the area if available (e.g. refugee setting vs. other), and duration of reusable pad use. We intended to assess publication and small-study bias by visual inspection of funnel plots for the outcome of use or uptake of RMPs, but studies were not sufficiently homogeneous for these procedures [5]. We intended to do a sensitivity analysis of low versus moderate-to good quality studies, as determined by the quality assessment; however, there was too little variety in the study quality to conduct this as planned.

Methods qualitative analysis

We followed the Critical Appraisal Skills Programme tool to examine the quality of the studies [6]. While the tool does not provide a definitive score to judge studies on their quality and / or include or exclude them, it enables the reader to understand the rigour of each study and the transparency of reporting, thus facilitating judgement of quality and interpretation of results. Two reviewers (LM and GZ) assessed each study independently, and examined and summarised the findings from these studies in combination to ensure a more robust and comprehensive explanation than would occur if each individual study was considered in isolation [7]. The data were analysed using thematic synthesis as described by Thomas and Harden (2008) [8]. Separately, the 2 qualitative reviewers extracted the themes and subthemes, along with illustrative quotes where applicable, from a first paper, and repeated this process for additional papers until a complete table of themes and subthemes with quotes was devised. A comparison of both tables was made, and a single definitive table produced. From this, the reviewers identified key themes. These qualitative results were integrated with quantitative results in the results section and are also available on page 12 of the supplement.

Methods visibility and availability

To assess visibility of the reusable pads, we used websites of governments and non-governmental organizations to obtain education materials to prepare girls for menarche from countries where the review-authors were familiar with the language (English, German, French, Spanish, and Portuguese; conducted by NJ, GZ, & AMvE). Search terms for this part included a country name, "menstruation", and "information", separate and in combination with "adolescent girls", and terms were translated where applicable. Websites from commercial providers of menstrual items were excluded, where identified. We compiled if sanitary pads, tampons, menstrual cups and reusable menstrual pads were reported in the material.

To assess the availability and price of reusable pads, we used web sources, e.g. listings of best reusable pads, to find websites of brands. Each individual brand was searched to identify its availability for purchase, through the web or otherwise, materials used, costs, lifespan and wash instructions. If a range of years was reported for lifespan (e.g. 3-5 years), we would use the midpoint (e.g. 4 years). We tried to determine where the pad was made and where it was sold; if it was available for sale in a retail store or if it would be purchasable directly through their website or an intermediary ecommerce website locally. Prices reported in local currencies were converted to United States Dollars with the applicable exchange rate on 21 December 2020. If a range of reusable pad products were available for purchase, we selected the day-time reusable pad model for a regular menstruation. If items were available for bulk purchase at a subsidized cost, we selected the purchase price when buying an individual unit. If individual units could not be purchased, we divided the bulk price by the number of units to calculate the individual unit cost. We also checked with experts to find out if there were reusable pad makers in developing countries which were missing on the list. However, because of language barriers we could not include producers from Asia. We noted a high turnover in producers and some producers only worked from websites that had log-in requirements, such as Facebook, Instagram or Linkedln; these were not included. Note that we did not search for period panties or other reusable externally applied menstrual items (e.g. labia pads); however, we included one article on period panties that otherwise met the inclusion criteria.

Methods for comparison of costs and waste

We used estimates on mean costs from previous calculations for disposable pads, tampons and menstrual cup [9]: the estimates for single-use pads and tampons were based on estimates from Amazon for 6 countries (United States, United Kingdom, India, Spain, China, Canada) in August 2018 and were 0.31 US$ for disposable pads, 0.21 US$ for tampons [9]. Based on a review of menstrual cup websites, we calculated as average 22.9 US$ for a menstrual cup [9]. We adjusted for inflation between August 2018 and December 2020 (https://www.bls.gov/data/inflation_calculator.htm). This changed the estimates to 0.32 US$ for disposable pads, 0.22 US$ for tampons, and 23.66 US$ for a menstrual cup. Because of the different numbers of RMPs that can be used in a menstruation, and the different lifetimes of the RMPs, we compared several options, and considered costs for RMPs separately for high and low-income countries (Table S12, Fig S5 and S6).

Changes with protocol

We screened the first 50 pages (500 entries) of Google Scholar. In addition, we screened the databases Science.gov and WorldWideScience to check if our information was complete. For quality assessments of included cohort studies, we used an adaptation of the Newcastle-Ottawa tool. Limitations of this tool were the absence of a clear non-exposure group and verification of exposure and outcome. In studies where experiences among RMP-users were compared with their previous experiences with other menstrual products, we assumed a before-after design, with a non-exposed group having the same characteristics as the exposed group. A study was considered moderate-to-good, if at least 4 criteria had been satisfied in the adapted Newcastle-Ottawa score (Table S1) or 5 in the Cochrane tool for trials. Because of the wide variability in types of reported outcomes, e.g. for leaking, we could not combine results from different studies using a risk ratio. We compared reported outcomes; however, given that most studies were "before-after design", using the same women reporting for experiences with a RMP and routine products,

the use of a chi-square test would have been inappropriate but insufficient information was available for a McNemar test. We intended to use the quality assessment as part of meta-regression and sensitivity analysis but decided not to conduct meta-regression because of the limited number of high-quality studies for meaningful comparisons, and the limited possibility of meta-analysis.

Table S1. Adaptation of quality assessment form for observational studies

	**Focus area**	**Category options†**
1	Representativeness of the exposed group<br><br>a) Is a description how women were recruited present?<br>b) If there is, are women representative of women in the community?	a) truly representative of women in the community (e.g. random selection in community) *<br>b) somewhat representative of the average woman in the community *<br>c) selected group of women (e.g. women who were enrolled from a clinic population)<br>d) no description of the derivation of the group<br><br>*Adaptation: 1 point if description present and women representative of women in the community (if not clear how recruited, or recruited from a school: 0 point)*
2	Selection of the non-exposed group	a) drawn from the same community/pool as the exposed group *<br>b) drawn from a different source<br>c) no description of the derivation of the non-exposed group<br><br>*Adaptation: If information is used from women before they were enrolled (e.g. a before-after study), or if there is control group that is coming from the same source as the exposed group: 1 point. Otherwise 0.*
3	Ascertainment of exposure	a) Objective measure of use<br>b) unsecure record<br>c) written self-report<br>d) no description<br><br>*Adaptation: If no objective measure: 0 point*
4	Comparability of exposed and unexposed group	a) differences examined and no differences reported in characteristics which are presented *<br>b) differences in characteristics present but no effect on outcome, or multivariate analysis for outcome available or randomized study *<br>c) differences in characteristic present, not shown if effect on outcome<br>d) no description/not examined<br><br>*Adaptation: If it is comparable to a before-after study or a comparison among characteristics of women who used and did not use the reusable pad and no differences, 1 point. If it is not present, 0 points*
5	Outcome assessment (leakage, frequency of change, vaginal inspection, bacterial cultures): not possible	a) independent blind assessment (observations or laboratory tests) *<br>b) record linkage (for observations or laboratory tests) *<br>c) not clear<br>d) no blind assessment (verbal or written reports)<br>e) no description<br><br>*Adaptation: if laboratory tests or inspection of vagina 1 point, if verbal report 0 points*
6	Attrition	a) complete - all subjects accounted for *<br>b) outcome not available for all subjects but unlikely to introduce bias - small number lost - <20%, or description provided of those lost *<br>c) outcome for less than 80% of people with exposure data and no description of those lost<br>d) no statement

* A study could be awarded a maximum of one star for each item, so in total 6 stars

2. Additional Results

Table S2. Search results databases from inception to 1 November 2020

	Source and website	Result (number)
1)	Pubmed<br>https://pubmed.ncbi.nlm.nih.gov	164
2)	Cochrane library<br>http://www.cochranelibrary.com	64
3)	Web of Science<br>https://webofknowledge.com	42
4)	Medline, Global Health and Cinahl through Ebscohost<br>https://www.ebsco.com/products/research-databases/global-health<br>https://www.ebscohost.com/nursing/products/cinahl-databases/cinahl-complete	134
5)	Science.gov<br>https://www.science.gov	460 in search, searched up to 180, 7 new
6)	WorldWideScience<br>https://worldwidescience.org	268: searched up to 210, 11 new
7)	Google Scholar<br>https://scholar.google.com	22 new
8)	Co-authors/reference lists/NGOs	8
9)	Manufacturer and User Facility Device Experience (MAUDE) from the US Food and Drug Administration (FDA), 10 year limit<br>(https://www.accessdata.fda.gov/scripts/cdrh/cfdocs/cfmaude/search.cfm)	0

Note: databases 5-7 were checked once the list from 1-4 was compiled. If there were 5 pages with no new material, we finished the search. Additional websites explored included International Menstrual Health Entrepreneurship (https://imher.net) and the Sustainable Sanitation Alliance (https://www.susana.org/en/), and the Menstrual Health Hub (https://mhhub.org/hive/research/all). Search terms: (((cloth* OR towel* OR pad OR suppl* OR product* OR absorbent*)) AND (((menses) OR menstrual) OR menstruation)) AND (((recyclable) OR reusable) OR sustainable)

Last search in FDA, February 2021

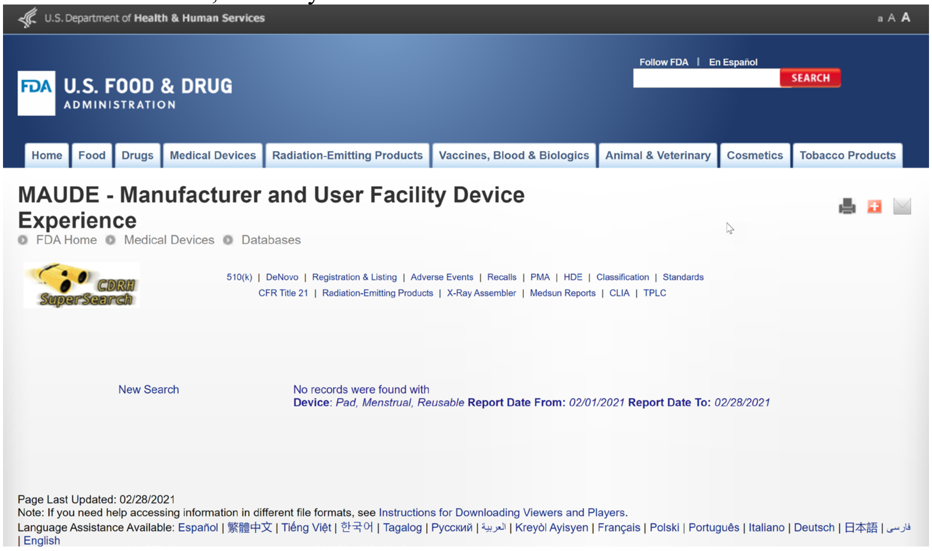

Table S3. Quality assessment of the studies involved (studies with quantitative information)

Table S3a. Trials

Study	Random-sequence generation	Allocation concealment	Blinding participants /staff	Blinding outcome assessment	Incomplete outcome data	Selective reporting	Other bias	Total
Montgomery 2016 [10] Hennegan 2016 [11, 12]	Not clear	Not clear	Not blinded	Not blinded	Outcome data for 580/1008 (57.5%) at 16 and 328/1008 (32.5%) at 24 months	Not detected	Enrolled non-menstruating women; errors in intervention delivery	Low-to-moderate 1
Garikipati 2019 [13]	Not clear	Not clear	Not blinded	Not blinded	453 identified, but only 293 baseline procedures; 277 outcomes at 6m (61.1% of total sample, 95% of consented sample)	Not detected	Needed educational intervention for acceptability reusable pad. Enrolled less than sample size required.	Low-to-moderate 2

Table S3b. Observational studies

Study	Participants representative of community	Non-exposed from same group	Ascertainment of exposure	Comparability of groups	Outcome assessment (observed/laboratory test vs. verbal/written)	Loss to follow up	Total	Type of Sample	Data collection
**Cohorts**									
Bardsley 2020 [14]	Not representative (students)	Yes (before/after study)	Verbal/written report	Same group	Verbal/written report	NA (retrospective cohort)	Low-to-moderate 2/6	Convenience sample	By female interviewer
Coker-Bolt 2017 [15]	Not representative (college students)	Yes (before/after study)	Verbal/written report	Same group	Verbal/written report	44/49 at follow up (90%)	Low-to-moderate 3/6	Convenience sample	Self-completed paper questionnaire
Femme International 2017 [16]	Not representative (schools and charities)	Yes (before/after study)	Verbal/written report	Same group	Verbal/written report	High (only 4-5% of initial sample)	Low-to-moderate 2/6	Convenience sample	Self-completed questionnaires
Gade & Hytti 2017 [17] (Womena)	Not representative (schools)	Yes (before/after study)	Verbal/written report	Same group	Verbal/written report	Attrition not reported	Low-to-moderate 2/6	Convenience sample	Trained female research assistants on mobile devices
Geismar 2018 [18]	Not representative (schools)	Yes (before/after study)	Verbal/written report	Same group	Verbal/written report	Attrition not reported	Low-to-moderate 2/6	Convenience sample	Unclear
IFRC 2016 [19] IFRC 2013 [20]	Representative (random from communities)	Yes (before/after study)	Verbal/written report	Same group	Verbal/written report	Loss to follow up unclear	Low-to-moderate 3/6	Stratified (by age) random sample	In Uganda and Somaliland by trained volunteers on mobile devices in local language. In Madagascar on paper
Kansiime 2020 [21] Nalugya 2020 [22]	Not representative (schools)	Yes (Before/after study)	Verbal/written report	Same group	Verbal/written report	188/232 at follow up (81%)	Low-to-moderate 3/6	Sample size calculation present	Self-completed on tablets
Kuncio 2018 [23] (UNHCR)	Not representative (schools)	Yes (Before/after study)	Verbal/written report	Same group	Verbal/written report	No clear loss to follow up	Low-to-moderate 3/6	Random sample but no calculation present	Trained female volunteers on mobile devices in local language
Mucherah 2017 [24]	Not representative (schools)	Yes (Before/after study)	Verbal/written report	Same group	Verbal/written report	Numbers not clearly presented	Low-to-moderate 2/6	Convenience sample	Self-completed questionnaire
Murthy 2015 [25]	Not representative (not random)	Yes (Before/after study)	Verbal/written report	Same group	Verbal/written report	Attrition not reported	Low-to-moderate 2/6	Convenience sample	Unclear
Hooper 2020 [26] Nabata & Clayton 2019[27]	Not representative (schools)	Yes (before/after study)	Verbal/written report	Same group	Verbal/written report	Attrition not reported	Low-to-moderate 2/6	Convenience sample	Unclear
Scott 2013 [28]	Not representative (schools)	Yes (Before/after study)	Verbal/written report	Same group	Verbal/written report	No clear loss to follow up	Low-to-moderate 3/6	Convenience sample	Trained female research assistants on mobile devices
**Surveys**						**Refusal rate**			
Amulen 2020 [29]	Not representative (schools)	Yes	Verbal/written report	Same group	Verbal/written report	Refusal rate not reported	Low-to-moderate 2/6	Sample size calculation Schools sampled by size	Trained interviewer in English, locally translated by assistant when necessary
Beksinska 2020 [30]	Not representative (higher education)	Yes	Verbal/written report	Same group	Verbal/written report	Refusal rate not reported	Low-to-moderate 2/6	Convenience sample of 500	Trained interviewers on paper or electronic (REDCap)
Borowski 2011 [31]	Not representative (recruited through web)	Yes	Verbal/written report	Same group	Verbal/written report	Refusal rate not reported	Low-to-moderate 2/6	Convenience sample	Self-completed online
Crankshaw 2020 [32]	Not representative (schools)	Yes	Verbal/written report	Same group	Verbal/written report	Refusal rate not reported	Low-to-moderate 2/6	Convenience sample	NR
Frank 2020 [33]	Not representative (transgenders)	Yes	Verbal/written report	Same group	Verbal/written report	Refusal rate not reported	Low-to-moderate 2/6	Convenience sample Snow-ball sampling	Interviewer
Goodson 2020 [34]	Not representative (schools)	Yes	Verbal/written report	Same group	Verbal/written report	Refusal rate not reported	Low-to-moderate 2/6	Convenience sample	Self-completed paper questionnaire

Hennegan 2020 [35]	Not representative (schools)	Yes	Verbal/written report	Same group	Verbal/written report	Refusal rate not reported	Low-to-moderate 2/6	Sample size calculation present	Self-completed paper questionnaire
Kim 2018 [36]	Not representative (unmarried women)	Yes	Verbal/written report	Same group	Verbal/written report	Refusal rate not clear	Low-to-moderate 2/6	Unclear	Unclear
Kuhlman 2019 [37]	Not representative (women in not-for-profit service organizations)	Yes	Verbal/written report	Same group	Verbal/written report	0 refusal rate	Low-to-moderate 3/6	Convenience sample	Interviewer administered surveys
Lamont 2019 [38]	Not representative (students)	Yes	Verbal/written report	Same group	Verbal/written report	Refusal rate not reported	Low-to-moderate 2/6	Convenience sample	Self-completed questionnaires on computer
Lenia 2019 [39]	Representative (random from communities)	Yes	Verbal/written report	Same group	Verbal/written report	Refusal rate not reported	Low-to-moderate 3/6	Sample size calculation present; stratified random sampling	Trained female research assistant
Miiro 2019 [40]	Not representative (schools)	Yes	Verbal/written report	Same group	Verbal/written report	2% refusal rate	Low-to-moderate 3/6	Convenience sample	Self-completed paper questionnaire in local language
Moon 2020 [41]	Not representative (university)	Yes	Verbal/written report	Same group	Verbal/written report			Sample size calculation present	Self-completed questionnaires online
Sivakami 2019 [1]	Not representative (schools)	Yes	Verbal/written report	Same group	Verbal/written report	Refusal rate not reported	Low-to-moderate 2/6	Sample size calculation present	Self- completed questionnaires
SNV 2014 [42]	Not representative (schools)	Yes	Verbal/written report	Same group	Verbal/written report	Refusal rate not reported	Low-to-moderate 2/6	Unclear	Unclear

Table S4. Manufacturers, city, country, and websites for reusable pads used in included studies

Reusable item	Manufacturer	City/country	Website
AFRIpad	AFRIpads (U) Ltd	Naguru, Kampala, Uganda	https://www.afripads.com/
Blumchen	Blumchen Stoffwindel GmBH	Grossmugl, Osterreich	https://stoffwindelcompany.at/Menstrual-care-98/
Days for Girls	Days for Girls International	Mt. Vernon, Washington, USA	https://www.daysforgirls.org/
KMET	Kisumu Medical and Education Trust	Kisumu, Kenya	https://kmetkenya.wordpress.com/2018/04/12/about-kmet-reusable-sanitary-towels/
Mwezi	Kenyan women's collectives	No address	No website
Safepad	Real Relief	Kolding, Denmark	https://realreliefwayhttps://www.realreliefway.com/life_saving_products/health/safepad™/safepad™
Shana pad	Kisumu Development Initiative	Kisumu, Kenya	No website
Subz pad	Subz Pants and Pads	KwaZulu-Natal South Africa	http://subzpads.co.za
Thinx period underwear	Thinx inc.	New York, NY, USA	https://www.shethinx.com
Uger pad	Jatan Sansthan	Rajasthan, India	http://jatansansthan.org/uger-pads-2/

Reusable pads or kits in alphabetical order

Table S5. Studies with other types of information contributing to the Reusable Menstrual Pad review (alphabetical order)

Study	Material	Country	Time & Population	Topic
Tang 2019 [43]	Journal Article	South Korea	Time NR. 9 disposable pads, 3 RMPs	Characterize the direction of liquid transport of sanitary napkin under different pressure loadings using a measurement method originated from Forced Flow Water Transporter Tester
Tang 2020 [44]	Journal Article	South Korea	Time NR. 9 disposable pads, 3 RMPs	Assess frictional force for sanitary pads using a modified Textile Stickiness Measurement System
Yilmaz 2019 [45]	Journal Article	Nepal	2016, 169 schoolgirls	The role that physical presence/proximity and ability to touch objects has on incentives for consumers to purchase goods, captured through willingness to pay (WTP) figures
Sundberg & Anderhell 2019 [46]	Thesis	Kenya	2019, 4 spacepads and 4 cotton RMPs	Simulate a menstruation to analyse microbial activity in reusable cotton and polyester pad
Tiku 2020 [47]	Preprint	Ethiopia	Time NR, population not applicable	Describes process of development of Reusable Menstrual Pad of cotton
Findlay 2012 [48]	Thesis	South Africa	Time 2018-2020, population not applicable	Describes process of development of Reusable Menstrual Pad from silicone

Section 2.1. Qualitative studies

Table S6. CASP-based appraisal of qualitative studies*

Authors	Clear statement of aims	Qualitative method appropriate	Design addresses aims	Recruitment appropriate	Data collected to address issue	Relationship considered	Ethical issues considered	Data analysis rigorous	Clear statement of findings	How valuable is research	Overall quality
Amulen 2019 [29]	Yes	Yes	Yes	Minimal	Satisfactory	No	Yes	Satisfactory	Yes	Medium	Medium
Bardsley 2020 [14]	Yes	Yes	Yes	Minimal	Satisfactory	Yes	Yes	Yes	Yes	Strong	High
Crofts & Fisher 2012 [49]	Yes	Yes	Minimal	No	Yes	Minimal	Minimal	No	No	Weak	Low
Gade & Hytti 2017 [17]	Yes	Yes	Yes	No	Yes	Minimal	Minimal	Satisfactory	Yes	Medium	Medium
Gaybor 2019 [50]	No	Yes	Yes	No	Minimal	No	No	Minimal	Yes	Weak	Low
Geismar 2018 [18]	No	No	No	No	Minimal	Yes	Yes	No	Minimal	Weak	Low
Goodson 2020 [34]	No	No	No	No	No	Researcher bias evident	Yes	No	No	Weak	Low
Hennegan 2017 [51]	Yes	Yes	Yes	Yes	Yes	Yes	Satisfactory	Yes	Yes	Strong	High
IFRC 2016 [19]	Yes	Yes	Yes	Yes	Yes	No	No	Minimal	Minimal	Medium	Medium
Jewitt 2014 [52]	No	Yes	Yes	No	Minimal	No	No	No	Yes	Weak	Low
Kambala 2020 [53]	Yes	Yes	Yes	Yes	Yes	No	Yes	Yes	Yes	Strong	High
Kuncio 2018 [23]	Yes	Yes	Yes	Satisfactory	Yes	No	Minimal	No	Minimal	Weak	Low
Lenia 2019 [39]	Yes	Yes	Yes	Minimal	Yes	No	Yes	Yes	Yes	Medium	Medium
Nalugya 2020 [22]	Yes	Yes	Yes	Satisfactory	Yes	Minimal	Minimal	Yes	Yes	Medium	Medium
Ottsen 2020 [54]	Yes	Yes	Yes	No	No	Yes	Yes	Yes	Yes	Medium	Medium
Schmitt 2017 [55]	Yes	Yes	Yes	Yes	Yes	Yes	Minimal	Yes	Yes	Strong	High
Sommer 2018 [56]	Yes	Yes	Yes	Yes	Yes	Satisfactory	Minimal	Yes	Yes	Strong	High
VanLeeuwen 2018 [57]	Yes	Yes	Yes	Yes	Yes	Yes	Yes	Yes	Yes	Strong	High
Vora 2020 [58]	Yes	Yes	Yes	Satisfactory	Satisfactory	No	No	No	Yes	Weak	Low

*Judgement on the quality of qualitative studies was based around the CASP tool which uses a 3-point rating scale: 'Yes' 'Can't tell' 'No'. For the purpose of this research we attempted to provide further information to help assess quality rather than using the category 'can't tell'. Some of the studies provided little detail within a category, so the 'no' response was not deemed applicable, whilst others provided a reasonable amount of information, despite not covering all considerations required by the CASP. The former we assigned 'minimal' and the latter 'satisfactory'. We also appraised each of the studies overall quality using a 3-point scale 'low', 'medium' 'high' based on the previous 10 category assignment.

Table S7. Characteristics of qualitative papers.

	Studies	Material	Location and time (years)	RMP brand, recipients and duration of use	Design and comparison	Qualitative sample size	Information age in years	Data collection method	Analysis method	Outcomes	Quality score
1	Amulen 2019 [29]	Thesis	Uganda, Bidibidi, refugee setting, time NR	Brand not stated, schoolgirls, duration not clear	Mixed methods; no intervention	Qualitative sample NR, 7 adults in interviews	15+ for girls in FGDs, adults for interviews	7 FGDs, 7 IDIs	Deductive content analysis	Assess the utilization of modern reusable menstrual pads among the school going adolescent girls in refugee settlement	Medium
2	Bardsley 2020 [14]	Thesis	Thailand, Mae La refugee camp, 2019	Days for Girls kits provided in previous 18 months via NGOs to schoolgirls in boarding houses	Mixed methods; no intervention given	Qualitative sample NR	13-22	Semi-structured interviews, visual mapping and field observations	Holistic thematic analysis	Assess effect of DfG kits - which participants use it, why they use it and how the use of the kit affects their sense of confidence and agency	High
3	Crofts & Fisher 2012 [49]	Journal Article	Uganda, South-Uganda, not further described. Time NR	Afripad (10 schools); Makapads (4 schools); No support (4 schools)<br><br>Duration not stated	Mixed methods RMPs vs. makapads and no pads (controls)	134 schoolgirls (senior 2-4), 9 RMP business leaders, 12 school staff	Schoolgirls range 13-20<br>Adult stakeholders	Participatory activities, KIIs, 40 FGDs, observation 18 school facilities	Not stated	Explore the difficulties experienced by schoolgirls in Uganda in managing menstrual hygiene and investigate the extent to which low-cost sanitary pads are part of the solution.	Low
4	Gade & Hytti 2017 [17]	Report	Uganda, Rhino Camp Refugee settlement, 2017	Afripads received by 42 schoolgirls & 21 mothers /staff,<br>4 months.<br>Total sample:<br>64 schoolgirls, 31 mothers / guardians, 7 teachers/village workers.	Cohort, 4 groups:<br>-Afripads<br>-Rubycups<br>-Both<br>-Choice	15 for IDI, otherwise not clear	Schoolgirls: median 16<br>Mothers: median 29.5<br>Not reported for other groups	KIIs, 7 FGDs, IDIs, explorative meetings with stakeholders, menstrual diaries and calendars	Qualitative content analysis	Assess the acceptability of introducing menstrual cups and reusable pads and measure safe use and care of the reusable products	Medium
5	Gaybor 2019 [50]	Journal Article	Argentina, Buenos Aires, 2015-2018	Not reported	Mixed qualitative methods; no intervention	56 users of menstrual cups or reusable pads (numbers for each not provided)	Range 20-35	Semi structured interviews (n=24) and FGDs (n=4)	Not stated	Explore from the perspective of the users, how reusable menstrual management technology is about a process of co-construction of technology and users identity	Low
6	Geismar 2018 [18]	Thesis	South Africa, Durban, 2018	Subz (package of 2-3 underwear and 6-9 RMPs), schoolgirls (8th grade and high school), duration ≤1 year	Mixed qualitative methods	5 students in FGDs, 4 adults and 2 students in interviews, number NR for participant observation	Students 15-17, adults not stated	Participant observations, 6 semi-structured interviews (2 project staff, 1 teacher, 1 volunteer) and 1 FGD	Not stated	To evaluate Project Dignity's impact on students' educational experiences and menstrual health practices.	Low
7	Goodson 2020 [34]	Thesis	Kenya, Kibwezi, 2019	Not reported	Mixed qualitative methods; no intervention	10 schoolgirls (Grade 7-Form 4), 6 women from community	Not stated	Community Based Participatory Action Research and participatory asset mapping activities*	Not stated	Examine how the education received by the girls and women on their menstrual cycle has or has not facilitated freedom, justice, empowerment, and equality within the lives of these girls and women	Low

	Studies	Material	Location and time (years)	RMP brand, recipients and duration of use	Design and comparison	Qualitative sample size	Information age in years	Data collection method	Analysis method	Outcomes	Quality score
8	Hennegan 2017 [51]	Journal Article	Uganda, Kamuli district, 2014	Afripad, schoolgirls, 2 years	Controlled 4-arm study: (i) education; (ii) reusable sanitary pads; (iii) both; (iv) control (no education and no reusable pads)	27 menstruating girls across 8 study schools (14 used RMPs)	Range: 12-17	Semi-structured interviews	Framework approach and cross-case analysis	Describe and compare experiences of menstruation at the conclusion of a controlled trial of reusable sanitary pad and puberty education provision in rural Uganda	High
9	IFRC 2016 [19]	Report	Madagascar, Uganda, Somaliland 2014	Kits with reusable pads (Afripad or similar product), refugee camps and vulnerable populations 4 months	Cohort study 3-arms: Kit A: only disposable pads Kit B: reusable pads Kit C: reusable /disposable pads	Number of participants in qualitative studies not reported	Range: 12-50	FDGs, IDIs, direct observation, key information interviews	Thematic analysis	Improve dignity of women and adolescent girls during emergency situations and to provide further evidence for MHM Kits as global relief items	Medium
10	Jewitt 2014 [52]	Journal Article	Kenya, Kisumu, Year not reported	3 locally made RMPs: Mwezi towel, Shana pad, and KMET towel. Duration not clear	Mixed qualitative methods; no intervention	53 participants in 9 schools	7 teens (13-15), no age range reported for adults	17 semi-structured interviews, 7 FGDs, direct observation	Not stated	Elicit feelings and opinions of schoolgirls around menstruation	Low
11	Kambala 2020 [53]	Journal Article	Malawi, 8 districts, 2018	Not applicable	Exploratory qualitative study	80 girls, 61 women and 35 key stakeholders (teachers, community health workers, service providers)	10-18	46 IDIs, 35 key informant interviews, 11 FGDs	Thematic content analysis	To explore the acceptability of menstrual product interventions and to explore implementation issues and views regarding the effect of menstrual products interventions on girls and women	High
12	Kuncio 2018 [23]	Report	Uganda 2018 Refugee setting	Hygiene kits with Afripads, schoolgirls, 3 months	Pilot feasibility study. All girls received same intervention, no comparison	Not clear	13-20	4 FGDs, 8-10 girls each	Thematic content analysis	Test the appropriateness and acceptability of introducing reusable sanitary pads to schoolgirls in the refugee context	Low
13	Lenia 2019 [39]	Thesis	Uganda Year not reported Refugee setting	No particular brand; Afripad is reported, duration of use unknown	Mixed methods cross-sectional study; no intervention	58	15-49	FGDs (54 participants); KII (4 participants)	Thematic content analysis	Assess knowledge, perceptions and practices on MHM of women in refugee settlement	Medium
14	Nalugya 2020 [22]	Journal Article	Uganda, Entebbe, 2017-2018	Afripads, secondary school students, duration not reported	Pilot feasibility study. All girls received same intervention, no comparison	Qualitative subsample not reported	13-21	IDI (40 participants), 4 FGDs	Thematic content analysis	Describe the implementation of the intervention and assess possible mechanisms of action	Medium
15	Ottsen 2020 [54]	Thesis	India, Kherwara, Rajasthan 2019-2020	Blumchen, schoolgirls (2 girls given, 1 girl seen after 1 year who used it for 6 months)	Cohort study	23 (11 girls and 12 mothers)	15+ among schoolgirls, mothers not reported	Baseline FGD with 9 girls, FGD with 12 mothers, IDI at end line with 8 girls Participant observation	Thematic content analysis	Explore how appropriate Civil Society Based Interventions are in the Indian context, to contribute to the discussion on how to facilitate girls' menstrual Inclusion	Medium

	Studies	Material	Location and time (years)	RMP brand, recipients and duration of use	Design and comparison	Qualitative sample size	Information age in years	Data collection method	Analysis method	Outcomes	Quality score
16	Schmitt 2017 [55]	Journal article	Myanmar Lebanon 2015	No particular brand. Refugees. Duration unknown.	Mixed methods; no comparison group	173	14-49	17 KIIs, 2 participatory mapping activities (39 girls), 6 FGDs (117 girls and women).	Thematic content analysis	Identify key barriers to MHM among displaced females, and generate insights into the types and content of MHM guidance for emergency response	High
17	Sommer 2018 [56]	Journal article	Tanzania; Nyaragusu, Mtendeli, and Nduta refugee camps 2016-2017	MHM emergency tool kits with reusable pads. No particular brand. Refugees. Duration unknown	Implementation evaluation study: no comparison group	128	14-49	28 KIIs (21 staff, 7 displaced girls/women), FGDs (number unclear, 100 displaced girls/women)	Deductive content analysis	Systematically monitor and describe the implementation of the MHM toolkit in an emergency	High
18	VanLeeuwen 2018 [57]	Journal article	Greece, Ritsona, refugee setting 2017	Thinx period underwear. Refugees. Duration unknown	Mixed qualitative methods.	30 displaced women, 5 humanitarian staff	18-50	Semi-structured interviews (11 participants), 4 FGDs	Inductive analysis	To explore the hypothetical acceptability and potential utility of a reusable menstrual underwear product in a refugee setting	High
19	Vora 2020 [58]	Book chapter	UK, Bristol, time NR	No particular brand. Homeless women. Duration unknown	Phenomenological case study No intervention provided	40 women	No information	40 IDIs	Not stated	Offer insight to ways in which women experiencing homelessness understand and negotiate their menstrual bodies within contexts of limited financial and material resources.	Low

IDI, in-depth interview. FGD, focus group discussion. KII, key informant interviews. NR, not reported. RMP, reusable menstrual pad.
*These activities involved daily observations, repeated interviewing, fieldwork journaling, and group discussions

Table S8. Themes in qualitative studies

Topic	Findings	Evidence	Study
**Leakage**	Afripads superior to improvised products	No direct	Hennegan 2017 [51]
	(Judged on appearance only) Most women felt Thinx pants would not be sufficient for heavy bleeding and others questioned absorbent capacity altogether. However, they felt it would be useful for double protection – with a pad, when travelling / night time etc	*'It is very thin. I wonder how it can absorb anything. Maybe I can use it only on the first and last days of my period when I only have a little blood. In the middle days I have a lot of blood and maybe it cannot absorb a lot of blood."* [43 years old]<br>*"At night we cannot go to the toilet because it was far and it was very dark, and we had a lot of fear that our daughters would have a problem at night. It would be good to have this product and a pad and you can be sure that you will not bleed through your clothing.*" [30–50 years old]	VanLeeuwen 2018 [57]
	General reusables, no specific brand - Some recounted that initially they were afraid that RMMT would not successfully prevent leakages. Most of the participants first tested the RMMT inside their home and when they felt comfortable they started using them in public environments	No direct	Gaybor 2019 [50]
	General reusables, no specific brand – Majority of women and girls in pilot indicated preference for pre-made re-usable pads as they were less likely to leak. Also more absorbent than cloth for heavy bleeding.	*'sometimes if we don't have (reusable) pads, we can't move from one place to another. So we can't move from home, we can't go anywhere'* (Burundi woman)<br>*For those with a lot of blood, the reusables are better. They can change up to 4 times a day and those are much better'* (Burundi woman)	Sommer 2018 [56]
	New Afripads (or similar) needed to be thicker – but also stated the material is bulky except AFRIpads	No direct	IFRC 2016 [19]
	AFRIpads less leakage compared with usual product	No direct	Gade 2017 [17]
	Specifically, the Afri pads (reusable pads) were said to be a good absorbent in all FGDs. The women and girls reported that the Afri pads are heavy (i.e substantial)	No direct	Lenia 2019 [39]
	Participants liked reusable pads because they absorb more menstrual blood (no comparison group / no specific type)	*'I can't easily spoil my cloth and it takes a long period of time while am using it" (FGD [participant 11] primary 1)*	Amulen 2019 [29]
	Participants mentioned that the way the Days 4 Girls pad stays secure on the underwear and the option to stack liners contributes to their confidence during menstruation–they have much less fear around blood leaking.	"*My skirt or my pants are not dirty because I am more safe when I put on the DfGKit. I am not afraid to go out because I am wearing it. It is safe,it will catch my blood.*" (Kho Thu Lu student)	Bardsley 2020 [14]
	Shana pads are too thin / too small	No direct	Jewitt 2014 [52]
**Movement**	Some concerns Afripad moved on movement	*'when you put it on, sometimes when you are walking it keeps on moving that way and backwards. They can protrude behind or in front'*	Hennegan 2017 [51]
	Shana pads don't stay in place	'*They run away when we are playing*'	Jewitt (2014) [52]
	AFRIpads girls reported not worrying about them shifting around.	Felt 'free' and 'relaxed'	Kuncio (2018) [23]
	Subz pads gave confidence that they were not going to move around or drop because they were kept in place. Students expressed a sense of security due to the fact that Subz pads fasten directly onto the accompanying panties. However, in contrast focus group discussion revealed areas for improvement in both the design and delivery of Subz pads and panties. In terms of product design, focus group participants noted that the panties are "too light" and expressed apprehension that they would tear up and fall during the course of the day	'*I love [the Subz packs].. because this one has a panty and [a disposable pad]doesn't have it. And it help[s] us, because with that one you feel like it's going to fall...And this one, you feel like you are wearing just a panty'* (Londile, student)	Geismar (2018) [18]

**Comfort**	Afripads superior to improvised products	No direct	Hennegan (2017) [51]
	Thinx pants perceived as comfortable in relation to pads.	*'Pads are uncomfortable, these are a good idea'*. [43 years old]	VanLeeuwen (2018) [57]
	Afripads superior to usual products and freedom to play	No direct	Gade (2017) [17]
	Reusable pads are soft and comfortable (no type / comparison)	*'I like the reusable pad because it is comfortable and absorbs more blood,"* (FGD [participant 11] primary 1	Amulen (2019) [29]
	Because Days for Girls pads were comfortable they gave girls confidence in wearing.	"not hot, but cool, and also soft" (MM)	Bardsley (2020) [14]
**Washing - self**	General reusables, no specific brand – Need privacy and soap to keep hygienic	*'though girls were given pads, they needed to wash their fingers with soap and they needed a private clean environment with water and soap to be able to maintain hygiene during their period*s'	Nalugya (2020) [22]
**Washing - product**	Afripad – need soap to clean, would ask for money from boys to buy soap	*'I use omo detergent to clean the pad. When it gets finished I can ask for money to pay for more'*	Hennegan (2017) [51]
	Mwezi pads concerns around how and when they could discreetly be washed	No direct	Jewitt (2014) [52]
	Assumption only – Thinkx appeared easy to wash but concerns around washing generally in setting with poor sanitation infrastructure	*In the beginning [when living in tents] it would be hard to clean it because we did not have water and somewhere to dry it'*. [23 years old]<br>*'I do not have a lot of time to be washing these. It is better for me to use pads and throw them in the garbage'*	VanLeeuwen (2018) [57]
	General reusables, no specific brand - Difficult for women in IDP camps to sustain use of re-usables because of lack of soap, so more likely to prefer disposables	No direct	Schmitt (2017) [55]
	General reusables, no specific brand – where reusables were provided in camps without a supply of soap usage was hindered	No direct	Sommer (2018) [56]
	General reusables, no specific brand – difficult to wash in private, some dislike of washing blood	*did not like the reusable pads because of washing them… (Karen)*<br>*does not like the reusable pads because of having to wash the blood, felt it was unclean….(Theresa)*<br>*because she has no place to wash and dry them without everyone knowing about her time of the month (Eve)*	Goodson (2020) [34]
	Lack of privacy for washing (new AFRIpads or similar) presents difficulties. Also lack of water particularly during dry season.	No direct	IFRC (2016) [19]
	AFRIpads - Didn't have time to wash during exam time. Sometimes not enough soap or water to wash	No direct	Kuncio (2018) [23]
	AFRIpads easy to wash, particularly during rainy season, but took a lot of time. Lack of privacy at home makes washing difficult. Many reported not having sufficient access to soap to wash their pads.	No direct	Gade (2017) [17]<br>Lenia (2019) [39]

	General reusables, no specific brand – women and girls only willing to wash absorbents as long as spectators could be avoided due to secrecy, particularly from males.	*If not for washing, reusable menstrual pads are even better, the other one given by Oxfam is heavy and stays for long but the problem is just washing, it takes long to dry and yet now days there is no sun. Somehow it is not good to be washing those things all the time*" (FGD, women 36-45 years, Bidibidi)	Lenia (2019) [39]
	The women and girls reported that the Afripads only challenge was that it requires washing of blood which they detested.	*"I don't like the pink pad because I fear to wash my blood so when I have my money I buy the blue pads and use" (FGD [participant 8] secondary 2)*<br>*"There is only one bucket at home that we all use for bathing, so I first have to wait for everyone to finish bathing then I wash my pad in darkness such that they don't disturb me while am washing the pad*" (FGD [participant 8] in secondary 1)	Amulen (2019) [29]
	Some FGD participants maintained that they didn't like them (not named product) because they feared washing the blood. Also lack of soap and lack of buckets.<br><br>Girls described how they cleaned the Days 4 Girls kits in the laundry facilities at their schools and said that they felt confident with this process.	No direct	Bardsley (2020) [14]
	Participants' main point of dissatisfaction with Subz was the necessity of washing the used pads themselves. Though two of the interviewed students reported that they did not mind washing the pads, several focus group participants cited the "yuckiness"of washing the bloody pads as the main impediment to their consistent use of Subz products	No direct	Geismar (2018) [18]
	Some participants reported that they feel nauseated with the smell of blood when washing used pads. Reusable sanitary pads were mostly worn at home for easy cleaning and washing but when girls were at school, disposable pads are used because they are easy to dispose of and do not require a lot of water.	No direct	Kambala (2020) [53]
	Participants communicated the difficulty in the cleaning of reusable sanitary products due to their constant spatial mobility (for the 'rough sleepers' and the 'hidden homeless' who are constantly moving around the houses of friends and hostels) and the lack of privacy of shared cleaning facilities (for those in shelters).	No direct	Vora (2020) [58]
**Drying**	Concerns around drying Afripad in relation to privacy. If outside then hidden in bushes or on top of chicken houses. Sometimes hung inside	No direct	Hennegan (2017) [51]
	Mwezi pads concerns around how and when they could discreetly be dried	No direct	Jewitt (2014) [52]
	Shana pads take long to dry	*Like if you are at school, it takes some time to dry it and you find that someone else will pick it from where you have left it*	Jewitt (2014) [52]
	Assumption only – Thinx pants problem drying generally	*When you clean it, you cannot dry it during the winter*	VanLeeuwen (2018) [57]
	General reusables, no specific brand – in IDP environment women had diminished privacy for washing and drying reusables so had preference for disposables	No direct	Schmitt (2017)
	General reusables, no specific brand – both staff and women highlighted challenges drying menstrual materials in refugee camps because of lack of space and privacy	*'It is a challenge ever to be alone. Sometimes you find many people in the house, mother, father, sisters, brothers. It becomes very difficult to change'* (Congolese adolescent girl)	Sommer (2018) [56]
	Lack of privacy for drying (new AFRIpads or similar) presents difficulties	No direct	IFRC (2016) [19]
	Afripads – No concerns amongst girls in boarding schools in drying pads as they were all in the same situation, however during FGDs it became clear that because the girls would often not have time to wash the pads until after school (or when they came home from school close to sunset), if they used 2-3 pads during the day, they	'because we're all girls, we're all going through the same thing"	Kuncio (2018) [23]

	would not be dry for use by the next morning. They also mentioned that even with the full pack of AFRIpads, they didn't feel like 4 pads was sufficient to manage their periods, particularly during the rainy season when drying times were significantly longer		
	AFRIpads difficult to dry during rainy season. The participants hang their reusable pads outside and cover them with a piece of fabric, and at times they are hung in a private place inside. If it rains, the pads are often put inside the house, and the rain can cause it to take two days for the pads to dry.	No direct	Gade (2017) [17]
	General reusables, no specific brand – women and girls only willing to dry absorbents as long as spectators could be avoided due to secrecy, particularly from males.	*Can someone really openly wash and dry a sanitary material? If panties cannot be dried in the open, what about the materials used for blood? Because one, it is regarded as a taboo and otherwise, with some superstition things. That's why they do hide such things. But at least they do take care of them*" (KII Hygiene promoter, Bidibidi).	Lenia (2019) [39]
	One participant reported there was nowhere for her to hang out the reusable pad	No direct	Ottsen (2020) [54]
**Changing**	Assumption only – Thinkx pants might have problems with changing as you would also need to take trousers off	*'It is not convenient for during my period because you must change all of your underwear all the time'.*	VanLeeuwen (2018) [57]
	Many girls reported that they could easily change their AFRIpads in either "changing rooms" or bath/wash rooms available to them at school and that those facilities were adequate	No direct	Kuncio (2018) [23]
**Carrying Soiled item**	General reusables, no specific brand – Not clear what to do with used pads. Difficult changing and carrying home	*'We can't (change at school) because when you wear the reusable it is difficult to change here. There is nowhere to put the used pads. Because we come with one we are wearing and don't have a bag to put it in there may be smells*' [adolescent Burundi girl]	Sommer (2018) [56]
**Safety**	General reusables no specific brand – negated health risks such as TSS, UTI, rashes, allergies. In particular referral was made to use of Glyphosate in pads which was thought to be carcinogenic (one participant)	'*I tried all the brands, but I always had a skin rash. My former gynecologist prescribed me creams, but it always came back. I was desperate so I started to search online and I found a website of a woman talking about these issues. She was selling cotton-made reusable pads. I tried them without much hope, and they worked* […]. (Martha -FGD)<br>'*All my thoughts and conversations began running on one track. My experience has been so good with the reusable pads that I wanted other women to feel the same. Especially those who suffer from allergies to disposables, as I did* '(Elena, Personal Interview	Gaybor (2019) [50]
	General reusables no specific brand – reusables are safe	*Reusable pads are durable and have no side effects. My parents had no problem with them. They told me they were safe'*	Nalugya (2020) [22]
**Itching / burning / Infection**	A few girls reported burning sensation associated with extended wear of Afripads	No direct	Hennegan (2017) [51]
	Participants reported no itching compared to usual method	No direct	Gade (2017) [17]
	Infections like Candida due to poor menstrual hygiene was identified as being a key barrier towards the use of the modern reusable menstrual pads.	*"The most common infection which is got by these girls is candida. This usually occurs when they put on a pad which is not washed well and dump at the same time*" (KI from Secondary school 2)	Amulen (2019) [29]
	Some participants noted that the Days 4 Girls pads did not result in any itching unlike some disposable products	'*When I use the disposable pad, I feel that my blood doesn't come a lot and it is itchy. So I feel it isn't comfortable and it makes the blood not come a lot. But the DfG one, that's fine*'.(Htee Moo Traw student)	Bardsley (2020) [14]
	(Any) reusables need good conditions to effectively wash and dry so as to avoid infections.	"*If they are hanged in a dark place, flies breed on it and when a person uses them they might cause infections such as mauka [candida]*". (IDI, Community woman, Salima)	Kambala (2020) [53]

**Cost**	Afripads reduced financial burden	No direct	Hennegan (2017) [51]
	Mwezi pads saves money	*It is very good….Better than Always 'cos if you only have one packet of Always you use it in one month then it is over. You look for another way of getting another packet, so it is very expensive. And if you get this renewable you are well-off now'*	Jewitt (2014) [52]
	General reusables no specific brand – cheaper than disposables	"If she had an option, she would because she felt it would assist her financially." (Debra)<br>"the reusable sanitary pads are valuable to this community because girls do not generally have monies to buy disposable pads even though that might be their preference….' (Carol)	Goodson (2020) [34]
	Generally most women preferred washable pads (New Afripad (or similar) over disposable as they felt they were more economical and sustainable	No direct	IFRC (2016) [19]
	Despite the overwhelming preference for commercially available products, if a good quality reusable product (see image right) was available at an affordable price, many girls and women indicated that they would like to use these.	*It will be cheaper than pad because you only pay once but will reuse it. The pads are one use only and you will pay every month whereas the other one is pay only once"* (SI: FGD women, formal workplace, urban).	Natoli (2017) [59]
	One of the most common comments from students using the Days 4 Girls pads related to financial benefits. Having the kit meant they didn't have to continually purchase disposable pads, thus saving money. Others reported they did not have any money to buy pads anyway.	*"I was happy when I gotit because it doesn't cost any money. This is for free and I can use it again and again"(Htee Moo Traw student)*<br>*'I feel that is it nice to use the DfG Kit because I can use it again and again after washing it. And it doesn't cost any money to buy. For the pad, we can't buy, we don't have money to buy pads' (IRC student)*	Bardsley (2020) [14]
	Student participants acknowledged financial difficulties of obtaining menstrual health products and the positive impact of Subz packs in this regard, saving the family a lot of money.	*'I feel happy about [the Subz pads], because [they] help us. I think that it would help me a lot because my mother sometimes do not have monies to buy for me, and [now] I get it in the school and I love it'* (Londile grade 8 student)	Geismar (2018) [18]
	The initial costs of purchasing reusable products were too steep for the women (rough sleepers / hostel dwellers etc), who possess limited financial resources	No direct	Vora (2020) [58]
**Underwear**	Afripads need fewer pairs of underwear compared to improvised products	No direct	Hennegan (2017) [51]
	Mwezi pads saves on underwear	No direct	Jewitt (2014) [52]
**Direct comparison**	Shana pad users less enthusiastic compared to Mwezi or K-MET pad users	No direct	Jewitt (2014) [52]
	Concept of Thinx accepted but preference to use customary products was commonly expressed	*There is no problem to use it, but I am accustomed to pads'*	VanLeeuwen (2018) [57]
	Adolescent girls in Singapore reported a preference for disposable pads compared to New Afripad (or similar)	No direct	IFRC (2016) [19]
	Overall participants who received both products (AFRIpad /Ruby cup related menstrual cup preference to convenience in terms of time used for cleaning and drying the products, time taken between changing/emptying the product, and overall potential duration of use.	'*When the period starts, I just put the menstrual cup and I use, and I don't spend much soap, there is no difficulties that I face compared to these other AFRIpads, it's just very simple, you enjoy, you move normal and you relax, no one will not know that you're in your periods that's the good thing'* (Mother School 1)	Gade (2017) [17]
	It was revealed that disposable pads were the most preferred menstrual absorbent by women and girls because they were easier to manage at a time when water and sanitation facilities were insufficient. They noted that disposable pads do not require washing	*The disposable pads are preferred by UNHCR because they are easier to manage compared to reusable pads. Before now, it was difficult to provide reusable pads since water was a challenge and the women lacked enough space managing the reusable pads as they often shared rooms with men. At least now the situation is stabilising*" (KII, UNHCR protection focal person Bidibidi).	Lenia (2019) [39]
	Generally, women and girls in all countries demonstrated preference for commercial products, with common reasons being: pads are more absorbent, they are more	No direct	Natoli (2017) [59]

	comfortable and stay attached to pants, they have a plastic layer to protect underpants, and they are easier to use when compared to washing and drying cloths.<br><br>Days 4 Girls pads were less itchy, cooler to wear and feel comfortable in comparison to disposables because they are more secure.<br>For the students, washing and drying the kits is easier than finding places to dispose of the used disposable menstrual pads in the refugee camp setting<br><br>Study concluded that overall, girls expressed that they prefer disposable pads over reusables. Preference for the reusable pads with both girls and women, depended on the type of materials that had been used in manufacture. Hard materials, such as cheap cottons, were reported to cause skin irritations and make it hard to walk especially when they become wet.	'*The kits are easier for me to use because the other pads are hot and move around'*.(Ko Thu Lu student) *'As for me, the pads are hot and it can be broken or got apart accidently that the heavy blood can flow outside of pads. Therefore, the kits are comfortable for me to use'*.(Ko Thu student)<br><br>No direct	Bardsley (2020) [14]<br><br>Kambala (2020) [53]
**Appearance**	Thinx pants were aesthetically pleasing, particularly in relation to pads	*although they are refugees, I do hear that they love to wear for their men, you know [...] because of the lace, it's very nice and attractive. [Humanitarian staff] We would need to use it with the same color and same style bra (group laughter) [30-50 years]*	VanLeeuwen (2018) [57]
**Environmental concerns**	General reusables, no specific product - Environmental concerns were brought up as another important reason for becoming a user of RMMT. Participants consider that following a certain lifestyle (zero-waste, responsible consumption) is a means to foster social change<br><br>Some ladies felt that reusable pads are preferable to disposable pads as they do not need to be disposed of.	*'It's easier to go out on the street on a protest and say I'm with the environmental cause, you know? But it is more challenging to live accordingly [...]. For me it was important to switch to reusables [...] what else can you do if you don't want to produce more waste*?<br><br>"*Disposable pads are just disposed of anyhow by the women and the girls in the community and burning of the pads is not good for the environment, burying the pads is also not good because there is plastic in the pads that take a long time to decompose, if disposed in a pit latrine, it get full fast and if disposed in a WC, it might get blocked*". (FGD participant, Community women, Salima).	Gaybor (2019) [50]<br><br>Kambala (2020) [53]

Due to the many differences in products and comparisons used, as well as the range of topics featured across the different studies, it was not appropriate to provide a measure of the strength of evidence for each outcome.

Section 2.2 Acceptability

Table S9. Reasons for not using reusable menstrual pads in surveys

Study	Reasons
Amulen 2019 [29]	Lack of soap, buckets. Fear to wash pads. No numbers provided
Lenia 2019 [39]	Disposable pads preferred by 60.0% (253/422)
Miiro 2018 [40]	When using single use pads less worry about leaks (233 or 76.1%), more comfortable (270 or 87.4%), easier disposable (227 or 72.5%), no need to wash/dry (257 or 84.0%), more modern (30.2%).<br>“When asked about the importance of attributes of sanitary protection, 316 (90.3%) reported disposable protection as important or very important, 170 (48.9%) washable protection as important or very important.”
Borowski 2011 [31]	Not likely to try an RMP due to washing (44.7% or 70/156)

Figure S1: Reported use of reusable pads in surveys in Ugandan schools, 5 studies, 2014-2019

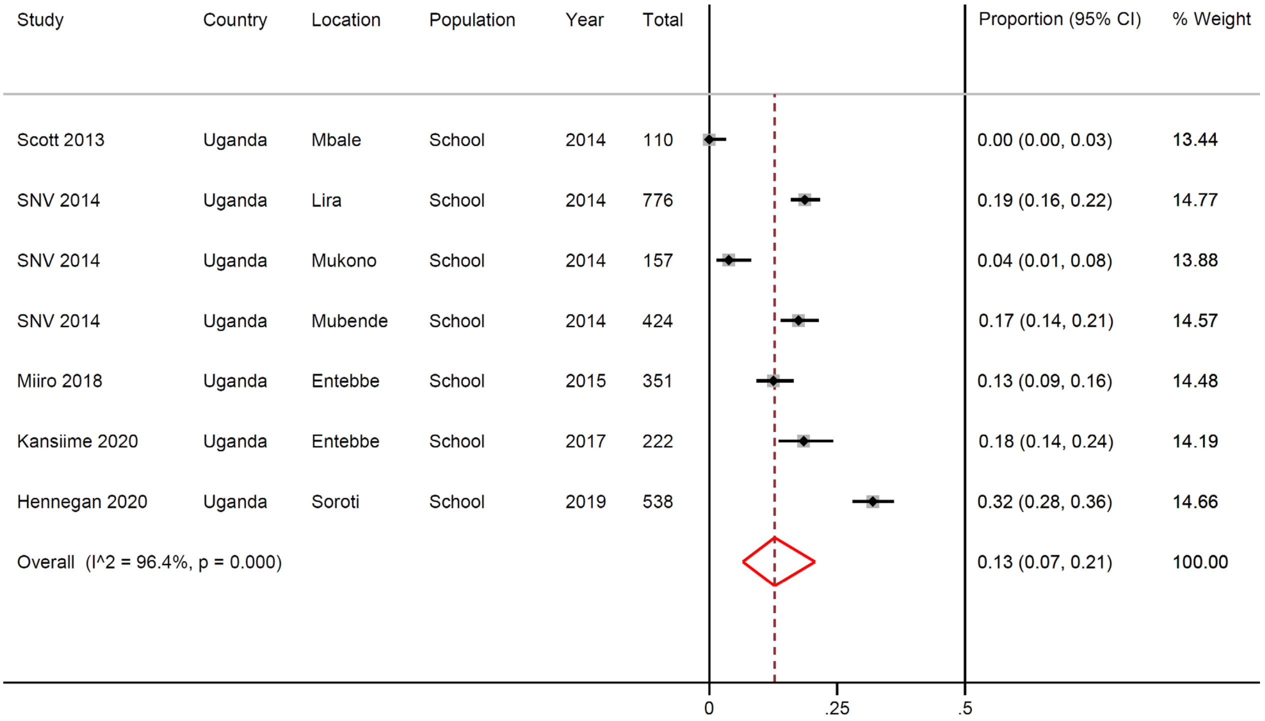

Notes: The studies presented provided information on use of RMPs in surveys or at enrolment in cohort studies.

Figure S2. Use of reusable pads by follow up time, 19 studies, 2014-2019

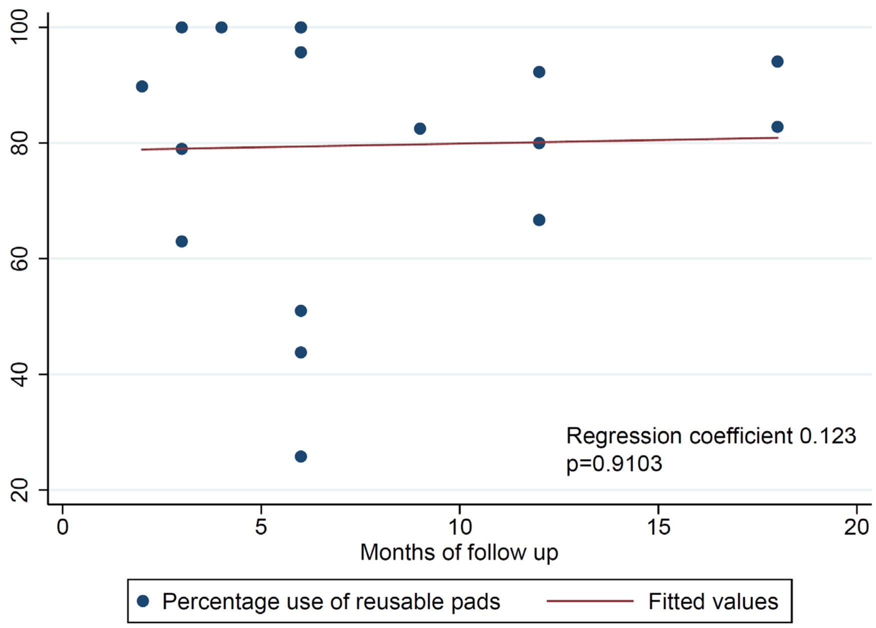

Figure S3. Uptake of reusable menstrual pads in schools by brand, Africa, 2014-2018

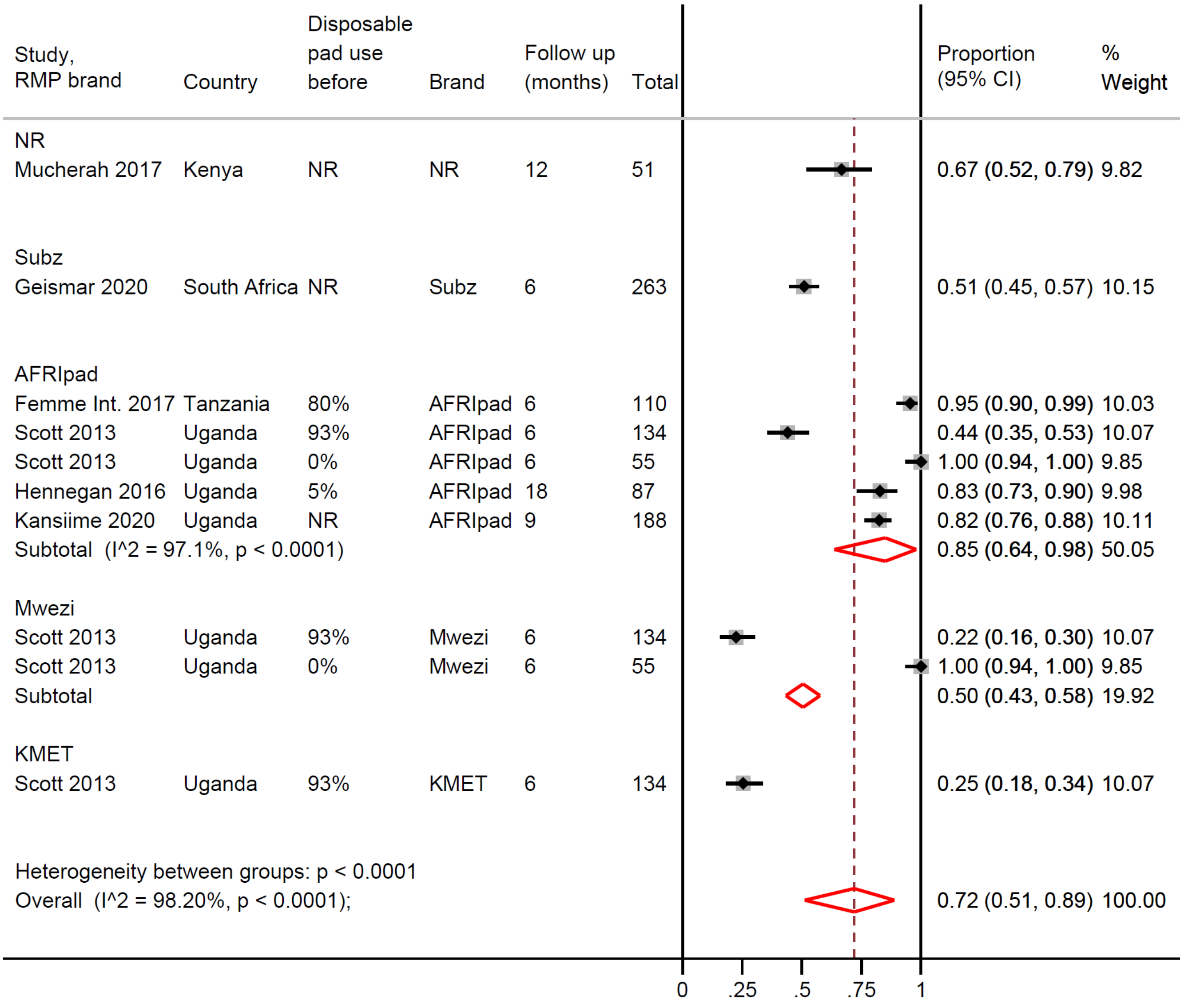

Section 2.3 RMP and education

Eleven studies evaluated the effect of RMPs on school attendance, compared to a control group or baseline conditions (Table 6). One trial used a school register, a potentially more objective measure of absence than self-reported absence; in this trial with three arms (one arm with education, one with RMPs and a combination arm), an increase in attendance was noted among RMP-users compared to control. However, results were inconsistent for the three arms; e.g. in the arm with education and RMPs, no increase in attendance was noted [10]. Most studies did not report a difference in school absence when using self-reported school attendance. A study in India noted a lower absence among RMP-users compared to users of cloths and disposable pads in Chhattisgarh, contrary to two other Indian states where higher absence was reported among RMP-users compared to users of disposable pads (no difference between RMP and cloth-users) [1].

Table S10. Effect of reusable menstrual pads on school attendance

<table>
<tr><th>Study</th><th>Country, study design, population, follow up time</th><th>RMP brand (number) & Alternative</th><th>RMP and attendance</th><th>Alternative item (or baseline) and attendance</th></tr>
<tr><td>Montgomery 2016 [10]</td><td>Uganda, trial, schoolgirls, 12-24 months</td><td>AFRIpad (6) and education vs. AFRIpad only vs. education only vs. none, trial</td><td>Afripads 5.23% (SD 29.48) increase in attendance (school attendance records) comparing baseline and endline (N=164, p=0.003 compared to control)</td><td>Education only: 1.46% decrease (SD 16.55) in attendance (p=0.045 compared to control)
Education and RMPs: 0.19% decrease (sd 27.66) in attendance (p=0.017 compared to control)
Control: 23.81% decrease (SD 34.82) in attendance</td></tr>
<tr><td>Hennegan 2016 [12]</td><td>Uganda, endsurvey trial, schoolgirls, 12-24 months</td><td>AFRIpads (6) vs. cloths</td><td>15% (11/72) RMP-users missed school (self-report: p=0.355* compared to cloth-users)</td><td>21% (23/111) cloth-users missed school</td></tr>
<tr><td>Bardsley 2020 [14]</td><td>Thailand, survey, schoolgirls, 18 months</td><td>Days for girls (8) vs usual item</td><td>66.2% (45/68) able to attend school more often with kit (self-report)</td><td>No baseline</td></tr>
<tr><td>Coker-Bolt 2017 [15]</td><td>Haiti, cohort, students, 2 months</td><td>Days for girls (8) vs usual item</td><td>No information</td><td>Baseline: 21.4% of participants ranked lack of feminine hygiene products as most important reason for school absence</td></tr>
<tr><td>Geismar 2018 [18]</td><td>South Africa, retrospective cohort, schoolgirls, 6 months</td><td>Subz (4) vs. usual item</td><td>61.2% (161/263): receiving Subz pack has made it easier to attend school during periods
48.7% (128/263): feel more confident in managing menstruation</td><td>Not reported</td></tr>
<tr><td>Kansiime 2020 [21]</td><td>Uganda, cohort, schoolgirls, 9 months</td><td>AFRIpad (4) at endline vs usual item at baseline</td><td>32.8% reporting missing at least one day of school (self-report, p=0.92 compared to baseline)
14.8% of 838 period days by 81 girls missed at endline (no p-value reported for comparison with baseline)</td><td>32.0% reporting missing at least one day of school (self-report)
14.6% of 554 menstruation days among 99 girls missed at baseline</td></tr>
<tr><td>Kuncio 2018 [23]</td><td>Uganda, cohort, schoolgirls, refugee camps, 3 months</td><td>Afripad (4) at endline vs. usual item at baseline</td><td>28.2% reported missing school during menstruation (no p-value reported compared to baseline, no denominators available)</td><td>50.0% reported missing school during menstruation</td></tr>
<tr><td>Mucherah 2017 [24]</td><td>Kenya, schoolgirls, cohort, 12 months</td><td>RMP (brand NR) and education (intervention school, cohort) vs none (control school). Evaluates perception of missing school, not missing school itself.</td><td>Likert Scale: 1 (strongle disagree) – 6 (strongly agree).
“If I miss school because of my period, I get lower marks or have a hard time catching up”: mean score 3.68, sd 1.12, n=34 (p<0.001† compared to control school)</td><td>Control school Likert Scale: 1 (strongle disagree) – 6 (strongly agree).
“If I miss school because of my period, I get lower marks or have a hard time catching up”: mean score 5.76, sd 0.58, n=91</td></tr>
<tr><td>Sivakami 2019 [1]</td><td>India, surveys Chhattisgarh, Maharashtra, Tamil Nadu, schoolgirls</td><td>RMP (brand NR) vs cloths & disposable pads</td><td colspan="2">Report of missing school during menstruation (self-report)
<table>
<tr><th></th><th>RMP (%)</th><th>Cloths (%)</th><th>p-value RMP vs. cloths*</th><th>Disposable pads (%)</th><th>p-value RMP vs. disposable pads*</th></tr>
<tr><td>Chhattisgarh</td><td>3/54 (5.6)</td><td>119/550 (21.6)</td><td>0.005</td><td>25/227 (11.0)</td><td>0.229</td></tr>
<tr><td>Maharashtra</td><td>36/304 (11.8)</td><td>11/100 (11.0)</td><td>0.820</td><td>23/385 (6.0)</td><td>0.006</td></tr>
<tr><td>Tamil Nadu</td><td>9/167 (5.4)</td><td>1/38 (2.6)</td><td>0.476</td><td>10/528 (1.9)</td><td>0.016</td></tr>
<tr><td>Total</td><td>48/525 (9.1)</td><td>131/688 (19.6)</td><td><0.001</td><td>58/1140 (5.1)</td><td>0.002</td></tr>
</table></td></tr>
<tr><td>Scott 2013 [28]</td><td>Uganda, secondary schoolgirls, cohort</td><td>KMET (6), AFRIpad (5), Homemade pad (MWEZI pad, 4)
(all received 10 disposable pads as well)</td><td>No evaluation available</td><td>Baseline: 27% (109/402) reported missing school due to menstruation (average 2 days/months); 90.5% (99/109) because of pain</td></tr>
</table>

*Chi-square test † t-test

Section 2.4 RMP development

Tang *et al*. (2019, 2020) compared nine disposable and three reusable pads in laboratory simulations. The three reusable pads were thicker and heavier and absorbed water slower compared to the disposable pads. They noted that in these laboratory models at 20 °C, the liquid transport properties of RMPs was poorer and the frictional force higher than the tested disposable pads [43, 44]. This may indicate that RMPs have wetter surfaces and a higher friction risk than disposable pads. However, it is not clear how well these laboratory models mimic menstruation; the materials used in RMPs may absorb better when the fluid is warm. The experiments were conducted at 20 °C whereas the body temperature in the female genital region is closer to 32 °C [60]. Additionally, menstrual blood may have different properties than the fluid that was used in the experiments. Four studies described the process of RMP development; two studies (in India and Ethiopia) described a 'standard' design using three layers and snaps around the underwear [25, 47]. An alternative South-African design (Nula) was a silicone holder and layer that stores blood and can be washed under the tap, dried, and reused [48]. Menstrual pads (reusable or disposable) do not need to be sterile [61]; however, wearing can lead to bacterial growth and this may lead to vaginal infections [62]. A polyester RMP design (Spacerpad) was compared against a cotton reusable pad for microbial activity under simulated conditions of menstruation, using a nutrient solution on a pad and incubation in an open plastic bag [46]. Both types of pads showed heavy growth of bacteria and fungi after about 8 hours incubation, which mostly disappeared after washing with soap [46]. In the web search for costs of pad, an RMP which claims to have antimicrobial properties (Safepad) was identified and may be of interest for further research and comparison [63]. For details about the brands, see table S4.

Example new pad development: Nula pad, made from silicone [48].

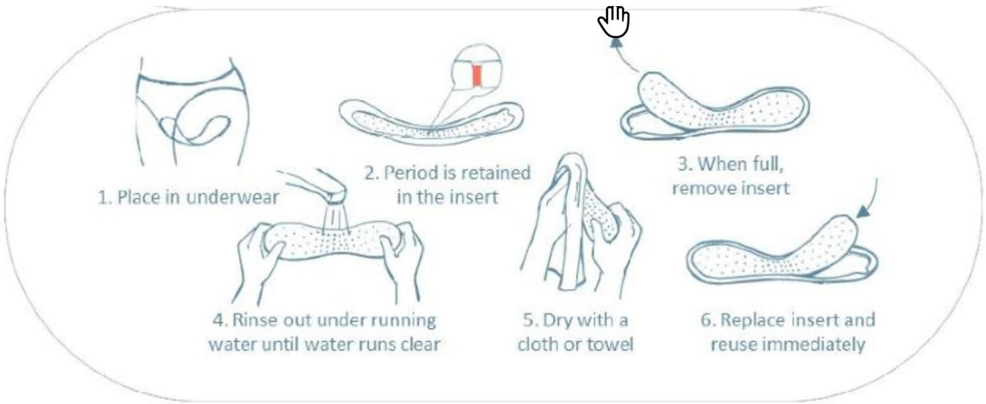

Section 2.5 Visibility, availability, costs and waste

Table S11. Studies with information on visibility or a potential interest in reusable menstrual pads

Study	Study design, time location, participants	Quote
Natoli 2017 [59]	Qualitative study, 2016-2017. Solomon Island (Guadalcanal), Fiji (Viti Levu/central), Papua New Guinea (Port Moresby). Focus group discussion, in-depth interviews, key informant interviews.<br>Girls in and out of school, women formal and informal employed, vulnerable women, vendor, teacher, health worker, leader, employer, n=307<br>No age information	"Interestingly, despite the overwhelming preference for commercially available products, if a good quality reusable product (see image right) was available at an affordable price, many girls and women indicated that they would like to use these. participants identified benefits in these reusable materials, citing the cost savings each month, the security of the pads (which fit into a moisture proof layer that clips onto underpants) and in the case of the Days for Girls kits, the colourful material that hides stains.<br>***"It will be cheaper than pad because you only pay once but will reuse it. The pads are one use only and you will pay every month whereas the other one is pay only once"*** (SI: FGD women, formal workplace, urban)**.**<br>Women felt that there would be opportunities for women's groups to sew and sell re-usable menstrual hygiene materials."
Kuhlmann 2020 [37]	Survey, 2019. USA (urban St. Louis). Secondary schoolgirls, n=59. Mean age 15.2, sd 1.2 years.	Nearly 40% indicated a desire to learn more about reusable pads/durable products. (30.5% regularly not enough money to by products for menstruation, 18/59)
Borowski 2011 [31]	Mixed study: survey and key informant interviews. 2011. 155 women. USA, web based. Age ≥18 years, 43.4% 25-34 yrs	"69% of the respondents were not aware of any green (eco-friendly) feminine hygiene products."<br>"When asked "*How likely would you be to try a reusable product that has to be washed or laundered to minimize your environmental and health risks?*" 44% of the women would not use such a product. However, the other 56% either use or would consider using such a product."
Garikipati 2020 [64]	Qualitative study: focus group discussions. 2017-2018. India (Hyderabad). Women. No age information.	"None of the women had heard of commercially made reusable cloth pads and very few had heard of other alternatives such as menstrual cups." "Fewer questions were asked about the re-useable cloth pad, and only in one FGD despite little awareness of them generally. These included how long it can be used for, whether it can be rewashed it in the bathroom and whether it would cause infection. The same issues were raised though in relation to whether the women might use in future – cost, comfort and health. Despite women's reluctance to wash traditional pieces of cloth, there was some interest in trying the re-usable pad. 'We will try for sure' (FGD2.5) 'what is the harm in trying? (FGD2.2) 'If we like then ok' (FGD2.2 and all agree)"
Moon 2020 [41]	Interviews with 14 young adults, online survey 150 young adults, intervention. South Korea	In terms of the method of menstrual education, participants expressed the desire to see menstrual products first-hand (n = 80, 53.3%) or audio-visually (e.g., a YouTube video; n = 79, 52.6%). Other preferred methods were slideshow presentations (n = 52, 34.7%), pamphlets (n = 43, 28.7%), face-to-face discussions (n = 21, 14%), and in-person lecture by menstruation experts (n = 20, 13.3%; statistics include duplicate responses).

Table S12. Menstrual items reported in education material for girls

Country	Website	Date accessed	Language	Disposable pads	Tampon	Menstrual Cup	Reusable pad
Africa, Asia, Central America	https://menstrualhygieneday.org/wp-content/uploads/2019/08/PSI_MHSRH_TechnicalBrief_v5_2019.pdf	16/06/2020	English	Yes	Yes	Yes	Yes
Africa-wide	https://www.afripads.com/	23/06/2020	English	No	No	No	Yes
Africa-wide	https://www.firstperiod.org/growing-up/	23/06/2020	English	No	No	No	No
Argentina	https://www.argentina.gob.ar/salud/glosario/ciclomenstrual	20/05/2018	Spanish	No	No	No	No
Argentina	http://www.msal.gob.ar/saludsexual/pdf/unfpa-baja.pdf (can't access try again)		Spanish	No	No	No	No
Argentina	http://www1.paho.org/hq/dmdocuments/2010/Recomendaciones-atencion-integral-salud-adolescentes-salud-sexual-reproductiva.pdf	21/05/2018	Spanish	Yes	Yes	No	No
Australia	https://www.betterhealth.vic.gov.au/health/conditionsandtreatments/menstrual-cycle	09/06/2020	English	Yes	Yes	No	No
Australia	http://www.cyh.com/HealthTopics/HealthTopicDetailsKids.aspx?p=335&np=289&id=1817#12	09/06/2020	English	Yes	Yes	No	No
Australia	https://www.healthdirect.gov.au/menstruation	09/06/2020	English	No	No	No	No
Australia	https://jeanhailes.org.au/health-a-z/periods/about-the-menstrual-cycle	09/06/2020	English	Yes	Yes	Yes	Yes
Australia	http://raisingchildren.net.au/articles/periods.html#sanitary	09/06/2020	English	Yes	Yes	No	No
Australia	https://brightgirlhealth.com/seminar-info/	23/06/2020	English	Yes	Yes	No	No
Brazil	http://bvsms.saude.gov.br/bvs/publicacoes/caderneta_saude_adolescente_menina.pdf	21/05/2018	Portuguese	No	No	No	No
Brazil	http://bvsms.saude.gov.br/bvs/folder/10006002035.pdf	21/05/2018	Portuguese	Yes	No	No	No
Canada	http://www.canadianliving.com/health/nutrition/article/stages-of-puberty	09/06/2020	English	Yes	Yes	No	No
Canada	http://www.cwhn.ca/en/node/44808	09/06/2020	English	Yes	Yes	Yes	Yes
Chile	https://www.clinicalascondes.cl/BLOG/Listado/Pediatria/La-primera-menstruacion	20/05/2018	Spanish	Yes	No	No	No
Chile	https://www.saludactual.cl/ginecologia/como-hablar-con-tu-hija-del-ciclo-menstrual.php	20/05/2018	Spanish	No	No	No	No
Colombia	https://www.minsalud.gov.co/Documentos%20y%20Publicaciones/Modelo%20de%20servicios%20de%20salud%20amigables%20para%20adolescentes%20y%20j%C3%B3venes.pdf	21/05/2018	Spanish	No	No	No	No
East Asia and Pacific (UNICEF)	https://menstrualhygieneday.org/wp-content/uploads/2016/12/Unicef_MHM_Good_PracticeGuidanceNote_EAPacific_2016.pdf	16/06/2020	English	Yes	Yes	Yes	Yes
Eritrea	http://www.wins4girls.org/resources/2017%20Eritrea%20MHM%20%20Teachers%20Booklet.pdf	16/06/2020	English	Yes	No	No	yes
Ethiopia	https://menstrualhygieneday.org/wp-content/uploads/2017/02/Growth_and_Changes_Ethiopia_Book_Tigregna.pdf	16/06/2020	English/Oromo (Ethiopian language)	Yes	No	No	No
France	http://www.doctissimo.fr/html/sexualite/hygiene-feminine/articles/se_7067_premieres_regles_panique.htm	23/07/2020	French	Yes	Yes	Yes	No
France	http://unesdoc.unesco.org/images/0022/002267/226792e.pdf	23/07/2020	French	Yes	Yes	Yes	Yes
Germany	http://www.netdoktor.de/Gesund-Leben/Womens-Health/Menstruation/Die-erste-Regel-Menstruation-527.html	23/07/2020	German	Yes	Yes	No	No
Ghana	http://www.wins4girls.org/resources/2017%20Ghana%20MHM%20education%20booklet.pdf	16/06/2020	English	Yes	No	No	No
Guatemala	http://www.osarguatemala.org/osartemporal/Archivos/PDF/201610/289_24.pdf (can't access try again)		Spanish	No	No	No	No
Hong Kong	https://www.studenthealth.gov.hk/english/health/health_se/health_se_pp.html (for parents)	09/06/2020	English	No	No	No	No
India	https://menstrualhygieneday.org/wp-content/uploads/2019/03/MoDWS-India_MGISC_Menstrual_Waste_Management_WASH_Network.pdf	16/06/2020	English	Yes	No	Yes	Yes
India	http://breakingthesilencereddroplets.com/index.aspx	23/06/2020	English	Yes	Yes	Yes	Yes
India	https://ecofemme.org	23/06/2020	English	No	No	No	Yes
India	https://www.your-life.com/workplace	23/06/2020	English				
Malawi	https://www.unicef.org/wash/schools/files/UNICEF-MenstrualHygiene-PRINT-27May15.pdf	09/06/2020	English	Yes	No	Yes	Yes
Mexico	http://www.saludnl.gob.mx/drupal/las-ni%C3%B1as-y-la-pubertad	20-May-18	Spanish	No	No	No	No
Netherlands	https://www.opvoeden.nl/puber/verzorging-en-veiligheid/lichaamsverzorging/maandverband-en-tampons/	23/07/2020	Dutch	Yes	Yes	No	No
Netherlands	https://rutgers.media/pubergids/ongesteld-worden/	23/07/2020	Dutch	Yes	Yes	Yes	Yes
Netherlands	https://www.seksualiteit.be/voorlichting/puberteit-en-lichaam/de-eerste-menstruatie	23/07/2020	Dutch	Yes	Yes	Yes	Yes
Netherlands	https://www.sense.info/ontdek-je-lichaam/meisjeslichaam/menstruatie/maandverband-en-tampons	23/07/2020	Dutch	Yes	Yes	Yes	No
New Zealand	https://www.healthnavigator.org.nz/health-a-z/m/menstruation/	09/06/2020	English	Yes	Yes	Yes	Yes
New Zealand	https://www.kiwifamilies.co.nz/articles/periods-information-sheet/	09/06/2020	English	Yes	Yes	No	No
Nigeria	http://www.wins4girls.org/resources/2017%20Nigeria%20Brochure%20Community%20Support%20in%20MHM.pdf	16/06/2020	English	Yes	No	No	No
Nigeria	http://www.wins4girls.org/resources/2017%20Nigeria%20Brochure%20Puberty%20growth%20and%20change.pdf	16/06/2020	English	Yes	No	No	No
Pakistan	http://www.wins4girls.org/resources/2017%20Pakistan%20Booklet%20for%20Students.pdf	21/06/2020	Urdu	No	No	No	Yes

Paraguay	http://www.paho.org/par/index.php?option=com_docman&view=download&category_slug=publicaciones-con-contrapartes&alias=384-manual-clinico-de-la-adolescencia&Itemid=253	21-May-18	Spanish	Yes	No	No	No
Peru	http://bvs.minsa.gob.pe/local/MINSA/1093_DGSP88.pdf	20-May-18	Spanish	No	Yes	No	No
Peru	http://sellomunicipal.midis.gob.pe/wp-content/uploads/2016/10/5_guia_facilitador_capacitacion_adoles_y_juventud_sexualidad.pdf	20-May-18	Spanish	No	No	No	No
Save the children	https://www.savethechildren.org/content/dam/global/reports/health-and-nutrition/mens-hyg-mgmt-guide.pdf	09/06/2020	English	Yes	Yes	Yes	Yes
Simavi (Africa and Asia)	https://simavi.org/wp-content/uploads/2018/10/MH-Manual-Digitaal-DEF.pdf	16/06/2020	English	Yes	Yes	Yes	Yes
Singapore	https://sg.theasianparent.com/your-daughters-first-period/	09/06/2020	English	Yes	Yes	No	No
Singapore	https://www.healthhub.sg/live-healthy/957/help-your-child-cope-with-puberty-and-self-esteem	09/06/2020	English	Yes	No	No	No
South Africa	https://www.health24.com/Lifestyle/Woman/Menstruation/Your-first-period-20120721	09/06/2020	English	Yes	Yes	No	No
South Africa	https://minacup.org/why-mina/	23/06/2020	English	No	No	Yes	No
South East Asia & Africa	https://www.susana.org/_resources/images/720/3-3865-7-1590669688.png	23/06/2020	English	Yes	Yes	Yes	Yes
Spain	https://www.sanitas.es/sanitas/seguros/es/particulares/biblioteca-de-salud/ginecologia/aparato-genital-femenino/sin012102wr.html	20-May-18	Spanish	No	No	No	No
Uganda	https://www.education.go.ug/files/downloads/Menstruation_Management_Reader_2017.pdf	09/06/2020	English				
UK	http://www.nhs.uk/Conditions/Periods/Pages/Introduction.aspx#Sanitary products	09/06/2020	English	Yes	Yes	Yes	No
UK	https://www.heygirls.co.uk/wp-content/uploads/2019/08/Hey-Girls-Schools-Guide.pdf	12/06/2020	English	Yes	Yes	Yes	Yes
UK	https://www.gov.uk/government/publications/period-products-in-schools-and-colleges/period-product-scheme-for-schools-and-colleges-in-england	12/06/2020	English	Yes	Yes	Yes	Yes
UK	https://menstrualhygieneday.org/wp-content/uploads/2017/09/FPA_periods-what-you-need-to-know.pdf	16/06/2020	English	Yes	Yes	Yes	No
UNICEF	https://www.unicef.org/wash/files/UNICEF-Guidance-menstrual-health-hygiene-2019.pdf	10/06/2020	English	Yes	Yes	Yes	Yes
Uruguay	http://www.inju.gub.uy/innovaportal/file/12678/1/guiasexualidadgeneroadolescentesfeb2011.pdf	21-May-18	Spanish	No	No	No	No
USA	https://www.acog.org/Patients/FAQs/Your-First-Period-Especially-for-Teens	09/06/2020	English	Yes	Yes	Yes	No
USA	https://www.everydayhealth.com/pms/a-teens-guide-to-her-first-period.aspx	09/06/2020	English	Yes	Yes	No	No
USA	https://www.girlshealth.gov/body/period/pads.html	09/06/2020	English				
USA	http://kidshealth.org/en/teens/menstruation.html	09/06/2020	English	Yes	Yes	Yes	No

Search term: [Country Name] menstruation information adolescents; Spanish: información para adolescentes menstru* [nombre del pais]; Netherlands: Menstruatie informatie meisjes; German: Menstruation Information madchen; French: Premiere Menstruation information fille Portuguese: informação para adolescents menstru*

† This pamphlet has information on the sponge for menstruation.

Disclaimer: This was a search to explore the mention of reusable pads as an option to deal with menstruation, and not intended to be fully inclusive of all potential websites. We do not endorse any website.

Figure S4. Menstrual items mentioned in education material on menarche, from 80 websites

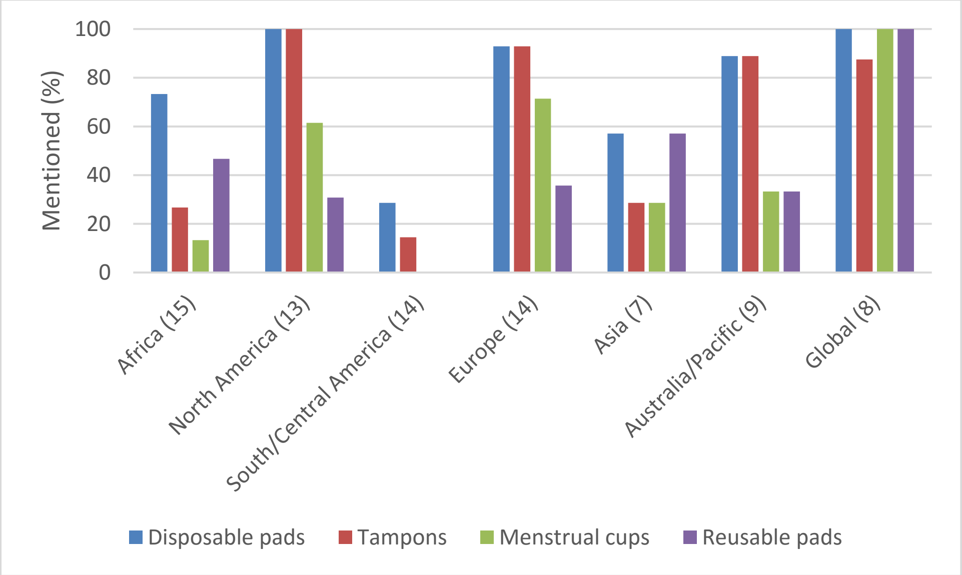

Global indicates education material targeted for more than one region.

For the methods see section 2; for a table of sources, see Table S12. The number of entries per region is in brackets. By region, disposable pads were mentioned in 60% or more except for South America (29%); tampons were only in Europe, North America and Australia/New Zealand over 80%, whereas menstrual cups were mentioned between 0% and 70.0% in regions. Reusable pads were mentioned between 0 and 57% in regions and 100% in materials covering more than one region. Overall, on 80 websites with educational materials on puberty and menarche, RMPs were mentioned as an option by 31 (39%), single-use pads by 61 (76%), tampons by 49 (61%), and menstrual cups by 33 (41%)

Table S13. Brands of reusable menstrual pads, price and lifespan estimate (where available)

Brand	Country	Price per unit (US$)	Lifespan estimate	Website
AFRIpads	Uganda	1	1+ years	https://www.afripads.com/
Agateka kit	Burundi	1	5 years	https://www.sacode.org/agateka-en
Eco Femme	India	3.18	3-5 years	https://ecofemme.org/shop/
Grace Pads	Malawi	3.75	1.5 year	https://www.gracepads.com/storewashdry
Kili pads	Tanzania	1.7	NR	http://www.tatuproject.org/kilipads/
Saukhyam reusable pads	India	1.26	4-5 years	https://www.saukhyampads.org
Subz	South Africa	2.76	3-5 years	http://subzpads.co.za
Supreme	Malawi	1.6	2 years	https://suprememalawi.com
Teamoy	China	2.83	NR	http://www.teamoy.com
Tina pads	Malawi		3 years	https://www.f6s.com/tinapads
Uger Pads	India		At least 60 washes	http://jatansansthan.org/uger-pads-2/
kMET pads	Kenya		1 year if properly cared for	https://www.doublexeconomy.com/post/kmet-s-comfort-pads-to-be-tested-in-study (Kisumu Medical and Education Trust)
Be Girl	USA	8	2 years	https://www.begirl.org/faq
Bluebird	Canada	20	NR	https://www.bluebirdpads.com
Blumchen	Germany	6.89	NR	https://stoffwindelcompany.at/Menstrual-care-98/
Charlie Banana	USA	6.33	2 years	https://www.charliebanana.com/collections/women-1/Feminine-Pad?rfsn=1458921.35457f
Cheeky wipes	UK	6.71	NR	https://www.cheekywipes.com/organic-cotton-cloth-sanitary-pads.html
Daisy & Bird	Australia	12.51	NR	https://www.daisyandbird.net
Days for girls	USA and Africa	1.50	3 years	https://www.daysforgirls.org/dfg-kits. Note: price depends on country. This price is for Kenya/Uganda
Domino Pads	USA	15.45	6 years	http://www.dominopads.com/pads.html
Duchess Reusable Sanitary Pads	USA	5.99	NR	https://www.dutchesscup.com/collections/all
Earthwise	UK	6.04	NR	https://earthwisegirls.co.uk/product-category/reusable-sanitary-towels/?cPath=1
Eco Rainbow	UK	9.06	NR	https://www.ecorainbow.co.uk
Ecodreams	UK	12.76	NR	https://ecodreams.co.uk/cloth-pads/
Fae pads	UK	7.38	NR	https://www.ecorainbow.co.uk
Fairy Hammocks	UK	6.04	NR	http://www.weenotions.co.uk/fairy-hammocks.html
Flo	Canada	6.04	NR	https://www.bummis.com/fabulous-flo-pads.html.html
Flowerchild Collective	Australia	11.37	NR	https://www.flowerchildcollective.com.au/category_3/Cloth-pads.htm
GladRags	USA	14.99	5 years	https://gladrags.com/
Gypsy Heart	Australia	11.17	NR	https://www.etsy.com/shop/GypsyHeartStore
H.E.R pads	Canada	14.33	NR	http://homesteademporium.com
Hannah pad	Australia	17.41	2-3 years	https://hannahpad.com.au
Heart felt	USA	4.2	NR	https://heartfeltusa.com/collections/reusable-menstrual-pads
Hesta/Rael organic pads	USA	12.33	up to 5 years	https://www.hestaorganic.com
Hibaby	USA	9.3	NR	Amazon
Honour your flow	UK	12.62	NR	https://www.honouryourflow.co.uk
lmse Vimse	Sweden	6.18	10 year warranty	https://imsevimse.co.uk/
Koree	UK	2.38	2 years	https://www.firstperiod.org
Kulmine	Germany	21.96	NR	https://www.purenature24.com/Kulmine-fabric-bandages-classic-soft-comfortable-and-does-not-apply-4674
Lady Pads	Germany	17.05	3-5 years	https://www.ladycup.eu/LadyPad.aspx
LadyDays	UK	11.41	NR	https://www.ladydaysclothpads.co.uk
Leekalos	Unclear	2.17	NR	https://www.amazon.com/Leekalos-Reusable-Menstrual-Pads-Incontinence
Lohan	USA	11.66	NR	
Lotus Blossum	USA	9.25	5 years	http://lotuspads.com/indexDefault.html
Luna wolf	UK	6.75	NR	http://www.weenotions.co.uk/category-163.html
Ma petite chou	UK	8.05	NR	https://www.twinkleontheweb.co.uk/acatalog/3-Ma-Petite-Chou-Upcycled-Bamboo-Menstrual-Pad-14MPC3.html

Mama's Cloth	USA	3.2	NR	http://www.bububibi.com/menstruals_pads.htm
Meow Meow	USA	12	NR	https://meowmeowtweet.com/collections/friends/products/menstrual-pads-1
Minivivi	UK	12.76	200 washes, around 4 years	https://minivivi.co.uk
Moonpads	Canada	12	10 years	http://newmoonpads.com/
My Cloth Fairy	Australia	9.35	5 years	https://www.facebook.com/myclothfairy/
New Moon	Canada	12	10 years	https://www.newmoonpads.com
Noonee Wilga	Australia	6.71	NR	http://www.nooneewilga.com
Obsidian Star	Australia	6.07	NR	http://www.obsidianstar.com.au/product-category/cloth-pads/
Paisley	Australia	13.27	NR	https://paisleypads.com.au/pages/about
Party in my pants	USA	12.99	5 years (65 washings or 5-7 years)	https://partypantspads.com/
PeaPods	UK	6.04	NR	http://www.weenotions.co.uk/peapods.html
Pleasure puss	Australia	6.05	3-5 years	http://pleasurepuss.com/
Popolini	Austria	21.96	NR	https://popolini.com/B2C/reinigen-und-pflegen/damenhygiene
Pride AF	Australia	7.58	NR	https://www.clothpadshop.com.au/store/pride-af/
Real Relief	Denmark	4.07	4 years	https://realreliefwayhttps://www.realreliefway.com/life_saving_products/health/safepad™/safepad™
Red Rags	UK	6.71	NR	https://redrags.co.uk
Ruby Love (previously pantyprop)	USA	9.75	NR	https://www.rubylove.com/about
Scarlet Eve	Australia	11.38	NR	https://www.scarleteve.com.au/collections/curvy-pads
Sckoon	USA	10.99	NR	https://www.sckoon.com/pages/sckoon-organic-cloth-menstrual-pads-faq
Sharkbite	USA	14	NR	https://www.etsy.com/shop/sharkbitepads
Smartliners	USA	3.5	NR	https://earthhero.com/products/beauty-care/smartliners-daily-reusable-menstrual-liners/
TCS Eco	UK	14.43	75 washes at least	https://www.tcs-eco.co.uk
Think Eco	Korea	8.33	up to 5 years	https://www.amazon.com/stores/think+ECO/page/290E2C3E-43FE-47AA-8745-31D5E38F37CC?ref_=ast_bln
Treehugger	Canada	NA	5-10 years	www.treehuggerclothpads.com
Wegreeco pads	USA	13.99	1-8 years	https://www.amazon.com/Wegreeco-Bamboo-Reusable-Sanitary-Pattern/dp/B07K68G39T?ref_=ast_sto_dp
Wemoon natural menstrual pads	Australia	12.86	5 years	https://wemoon.com.au/
Willowpads	USA	10.95	6 years	http://thewillowstore.com

Note: This is not an exhaustive list of all reusable menstrual pads available, just a snapshot of RMPs where we could find further information. There are more brands, e.g. in Asia that we could not include because of language issues.

Costs and availability: Summary of findings from websites
Because of limitations in our search and a high turnover or changing names of brands, we note that results of this search should only be considered as a snapshot or sample of what was available in the English language on the web in 2020. We identified at least 110 brands but could only access 73 websites. Thirteen were in LMIC and 61 in HMIC countries; it was not always clear where the RMPs were produced. Eleven were non-commercial (subsidized or not-for-profit); 9 of these were in LMIC. Most RMPs have three layers (soft top to the skin, absorbent material in the middle and bottom layer that may or may not be water resistant); cotton, bamboo and polyester were the most common used materials. Organic options were offered by 33 brands (44.6%). The most common design was the wing-shape that goes around the underwear and is fixed with one or two snaps (for an example see appendix pp); other designs include wingless pads that rely on firm underwear, winged holders and inserts, pads that snap to special underwear or pads with slings that can be used without underwear. An estimate of the lifetime of the RMP was found for 34 brands with a mean of 4.3 years (sd 2.3, median of 4 years, range 1-10 years). The mean estimated lifetime for brands in LMIC was 2.9 years (sd 1.4, median 3, range 1-5 years, n=11) and was less than the lifespan in HIC (mean 4.9, sd 2.4, median 3, range 2-10 years, n=23). For 58 RMPs, wash instructions could be identified (see below). Although most sites recommended to change as needed, depending on the blood flow, in LMIC the range of hours mentioned for wearing an RMP was 4-8 (n=5), whereas in HIC this was 2-6 hours (n=7). Some brands offered a ziplock bag (in LMIC) or more fancy bags (HIC) for carrying used RMPs. Brands from LMIC generally sold locally (in-country 8/13, 61.5%), whereas brands from HIC were more likely to sell globally (78.7%) (as much as could be identified). Most products were sold online, and the mention of shops was rare (2/74); in LMIC, door-to-door or local selling was mentioned for two brands. For 69 brands, we noted prices for an average product (medium or small daytime pad for regular bleeding); the mean price was $8.95 (sd $5.08, range $1.00-21.96, median $8.33, n=69). In LMIC this was $2.06 (sd $0.99, range $1.00-3.75, median $1.65, n=10) and in HIC $10.11 (sd $4.54, range $2.17-21.96, median $9.75, n=59). Many brands offered starter packs for girls reaching menarche, or value-packages with multiple pads or pads of different sizes, and savings calculators or calculations about savings were present on several sites (e.g. Partypants, Treehugger, Pleasurepuss, TCS-eco pad). Many sites offered additional products like period underwear, labia pads, reusable infant diapers or face masks. Some sites specialized in more gender-neutral products for transgender persons (Pride AF). In the studies in LMIC involved in this review, girls or women received 4-9 RMPs. Recommendations on number of pads needed in HIC are considerable higher, ranging from 6-27 RMPs per period, with different sizes of RMPs. We did not make an estimate of waste of RMPs, given the wide variability of materials used. It should be noted that many RMPs use polyurethane (PUL) in a layer at the back of the pad to prevent leaking, and this component is not compostable. Newer types of polyester, which are sometimes used, can be biodegradable, but that should not be taken for granted. One RMP (Safepad) claims to have antimicrobial properties to yeast, gram- and gram+ bacteria and fungi that withstand washing.

Washing of RMPs
Wash instructions could be found For 57 RMPs. For about half of them, soaking (2 to 30 to 60 minutes to 24 hours) or rinsing with cold water before washing was recommended to clean away the blood (hot water can stain the pad); or the pre-soak function on some washing machines could be used. RMPs can be washed by hand or in the washing machine with soap. They can be tumbled dry on low heat or dried in sunlight (although some instructions state not to put in direct sun). Some brands recommend not using the tumble dryer because this may be a risk of tearing. RMPs do not need to be washed separately from other clothes. When using the washing machine, conditioner, fabric softener, dryer sheets or bleach is not recommended. Recommended temperatures for washing machines range from cold 30-40 to 60 °C.

Several brands recommend washing before first use to provide better absorbtion. Shrinkage is normal for cotton in the first few washes. Ironing is not recommended, especially for the pads with a PUL layer at the back. Some makers recommend rinsing the pads while showering (by stepping on them). Some suggest there is no need for soaking (e.g. Tree Hugger: “keep in wet bag until washing in washing machine, wash in cold, and then in dryer”).

Table S14. Calculations of costs per sanitary item over 2.5 and 5 years

**Single-use pads**							
Number per month	4	8	9	12	**15**	20	25
Costs per item (US$)			0.32	0.32	**0.32**	0.32	0.32
Number per year			117	156	**195**	260	325
Cost per year (US$)			37.44	49.92	**62.4**	83.2	104
Costs per 2.5 years (US$)			93.6	124.8	**156.00**	208	260
Waste per 2.5 years (number)			292.5	390	**487.5**	650	812.5
Costs per 5 years (US$)			187.2	249.6	**312.00**	416	520
Waste per 5 years (number)			585	780	**975**	1300	1625
**Tampons**							
Costs per item (US$)			0.22	0.22	0.22	**0.22**	0.22
Number per year			117	156	195	**260**	325
Cost per year (US$)			25.74	34.32	42.9	**57.2**	71.5
Costs per 2.5 years (US$)			64.35	85.8	**107.25**	**143**	178.75
Waste per 2.5 years (number)			292.5	390	487.5	**650**	812.5
Costs per 5 years (US$)			128.7	171.6	214.5	**286**	357.5
Waste per 5 years (number)			585	780	975	**1300**	1625
**Reusable menstrual pad, low- and middle-income countries, lifetime 2.5 years**							
Costs per item (US$)	2.06	**2.06**	2.06	2.06	2.06	2.06	2.06
Number per year	4	**8**	9	12	15	20	25
Cost per year (US$)	8.24	**16.48**	18.54	24.72	30.90	41.20	51.50
Costs per 2.5 years (US$)	8.24	**16.48**	18.54	24.72	30.90	41.20	51.50
Waste per 2.5 years (number)	4	**8**	9	12	15	20	25
Costs per 5 years (US$)	16.48	**32.96**	37.08	49.44	61.80	82.40	103.00
Waste per 5 years (number)	8	**16**	18	24	30	40	50
**Reusable menstrual pad, high income countries, lifetime 5 years**							
Costs per item (US$)	10.11	**10.11**	10.11	10.11	10.11	10.11	10.11
Number per year	4	**8**	9	12	15	20	25
Cost per year (US$)	40.44	**80.88**	90.99	121.32	151.65	202.20	252.75
Costs per 2.5 years (US$)	40.44	**80.88**	90.99	121.32	151.65	202.20	252.75
Waste per 2.5 years (number)	4	**8**	9	12	15	20	25
Costs per 5 years (US$)	40.44	**80.88**	90.99	121.32	151.65	202.20	252.75
Waste per 5 years (number)	4	**8**	9	12	15	20	25
**Menstrual cup**	One-time expenditure of 23.66 US$, good for 10 years						

Examples:
If a woman in an LMIC used 8 RMPs with a life span of 2.5 years instead of 15 single-use pads per menstruation, she would spend 16.48 US$ and save approximately 140 US$ and the waste of ~488 single-use pads in 2.5 years; over 5 years this would amount to 280 US$ saved and the waste of close to 1000 single-use pads. Note that with a life span of 2.5 years, every number of RMPs examined (4-25 RMPs per period) was always cheaper than the lowest number of single-use pads per menstruation (9 single-use pads per 2.5 years would cost 93.60 US$; 25 RMPs with life span 2.5 years would cost 51.5 US$).
Even 25 RMPs with a life span of one year is cheaper than 15 single use pads per month.

If a woman in a HIC used 8 RMPs with a life span of 5 years instead of 20 single-use tampons per menstruation, she would spend 81 US$ and save ~ 62 US$ and the waste of 650 single-use tampons over 2.5 year; over 5 years it would save her approximately US$ 205 and the waste of 1300 single-use tampons. Note that up to 12 RMPs in would still be cost-saving in HIC, compared to 20 single-use tampons per period.
In bold and colour in the table the numbers that were used for these examples: red for LMIC and green for HIC.

Figure S5. Estimation of costs of different products used for menstruation over 2.5 and 5 years compared with RMPs with a life span of 2.5 years in LMIC and 5 years in HIC

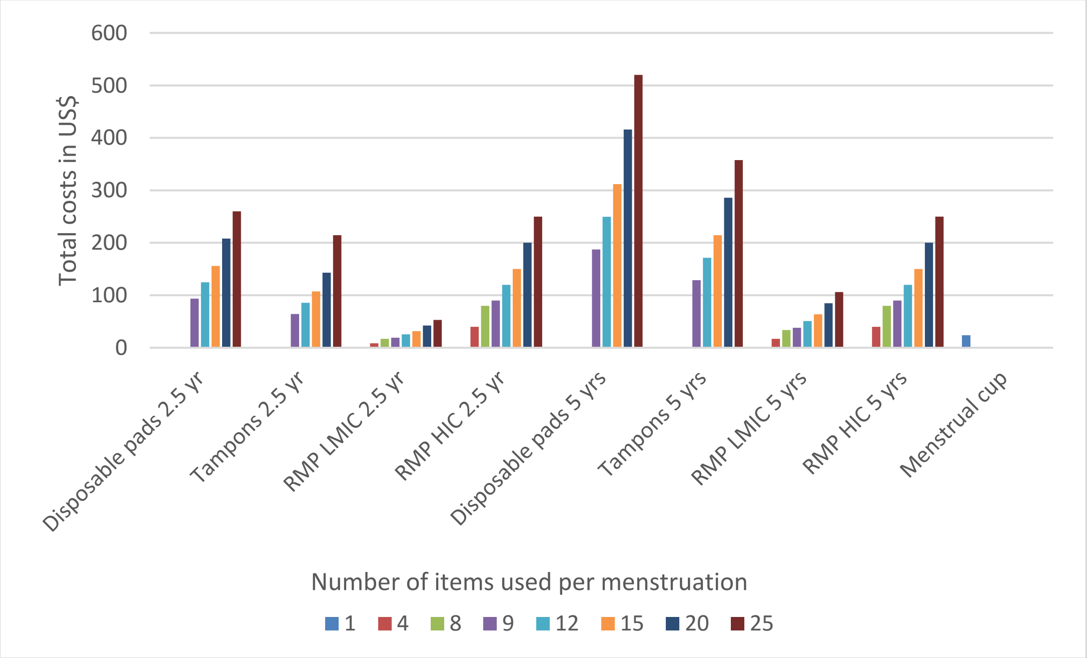

RMP, reusable menstrual pad. LMIC: low- and middle-income countries. HIC: high income countries
Combined use of menstrual products during a period (e.g. panty liners for light days), inflation, production costs and opportunity costs were not considered in these calculations.

Figure S6. Estimation of waste of different products used for menstruation over 2.5 and 5 years

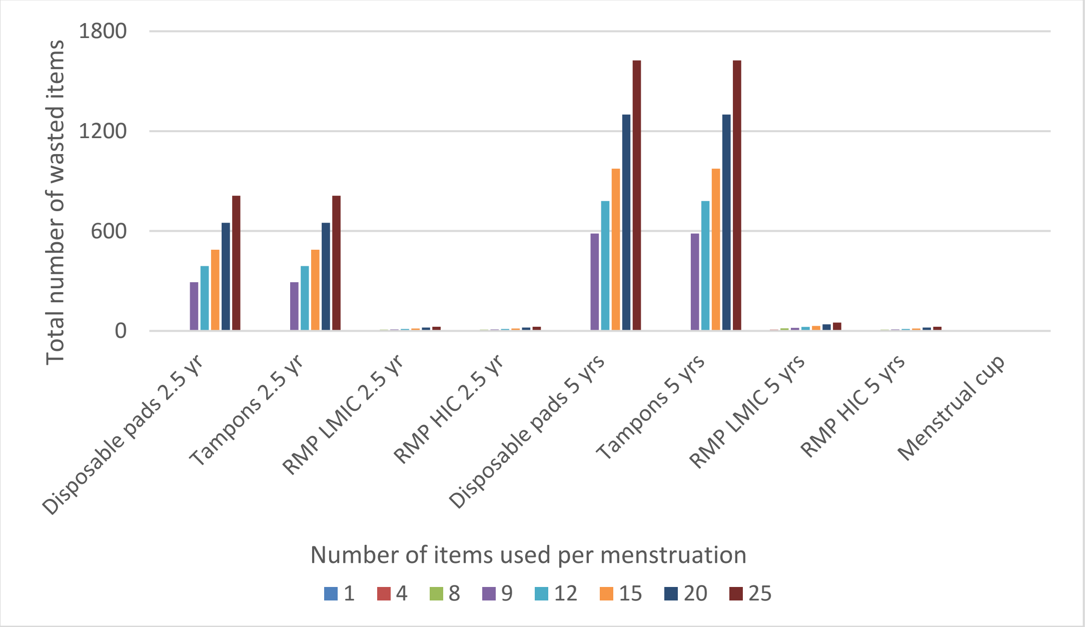

RMP, reusable menstrual pad. LMIC: low- and middle-income countries. HIC: high income countries

References

1. Sivakami M, Maria van Eijk A, Thakur H, Kakade N, Patil C, Shinde S, et al. Effect of menstruation on girls and their schooling, and facilitators of menstrual hygiene management in schools: surveys in government schools in three states in India, 2015. J Glob Health. 2019;9(1):010408. Epub 2018/12/14. doi: 10.7189/jogh.09.010408. PubMed PMID: 30546869.
2. UNICEF. Guide to menstrual hygiene materials 2019 [27 July 2021]. Available from: https://www.unicef.org/wash/files/UNICEF-Guide-menstrual-hygiene-materials-2019.pdf.
3. Higgins JP, Thompson SG, Deeks JJ, Altman DG. Measuring inconsistency in meta-analyses. BMJ. 2003;327(7414):557-60. doi: 10.1136/bmj.327.7414.557. PubMed PMID: 12958120.
4. Deeks JJ, Higgins JP, Altman DG. Analysing data and undertaking meta-analyses. In: Higgins JP, Green S, editors. Cochrane handbook for systematic reviews of interventions (version 510): The Cochrane Collaboration; 2011.
5. Public Health England. PHE Technical guide- Funnel plots 2018. Available from: https://fingertips.phe.org.uk/profile/guidance.
6. Critical Appraisal Skills Programme (CASP). CASP Checklists 2017. Available from: http://www.casp-uk.net/casp-tools-checklists.
7. Dixon-Woods M, Agarwal S, Jones D, Young B, Sutton A. Synthesising qualitative and quantitative evidence: a review of possible methods. J Health Serv Res Policy. 2005;10(1):45-53. Epub 2005/01/26. doi: 10.1177/135581960501000110. PubMed PMID: 15667704.
8. Thomas J, Harden A. Methods for the thematic synthesis of qualitative research in systematic reviews. BMC Med Res Methodol. 2008;8:45. Epub 2008/07/12. doi: 10.1186/1471-2288-8-45. PubMed PMID: 18616818.
9. van Eijk AM, Zulaika G, Lenchner M, Mason L, Sivakami M, Nyothach E, et al. Menstrual cup use, leakage, acceptability, safety, and availability: a systematic review and meta-analysis. Lancet Public Health. 2019;4(8):e376-e93. Epub 2019/07/22. doi: 10.1016/S2468-2667(19)30111-2. PubMed PMID: 31324419.
10. Montgomery P, Hennegan J, Dolan C, Wu M, Steinfield L, Scott L. Menstruation and the Cycle of Poverty: A Cluster Quasi-Randomised Control Trial of Sanitary Pad and Puberty Education Provision in Uganda. PLoS One. 2016;11(12):e0166122. Epub 2016/12/22. doi: 10.1371/journal.pone.0166122. PubMed PMID: 28002415.
11. Hennegan J, Dolan C, Wu M, Scott L, Montgomery P. Schoolgirls' experience and appraisal of menstrual absorbents in rural Uganda: a cross-sectional evaluation of reusable sanitary pads. Reprod Health. 2016;13(1):143. Epub 2016/12/08. doi: 10.1186/s12978-016-0260-7. PubMed PMID: 27923379.
12. Hennegan J, Dolan C, Wu M, Scott L, Montgomery P. Measuring the prevalence and impact of poor menstrual hygiene management: a quantitative survey of schoolgirls in rural Uganda. BMJ Open. 2016;6(12):e012596. Epub 2017/01/01. doi: 10.1136/bmjopen-2016-012596. PubMed PMID: 28039290.
13. Garikipati S, Docherty RJ, Phillips-Howard PA. What's the bleeding problem? Policy and attitudes towards sustainable menstrual hygiene materials in India Liverpool, UK: University of Liverpool, Management School; 2019. Available from: https://ideas.repec.org/p/liv/livedp/201907.html.
14. Bardsley TM. Understanding the impacts of humanitarian menstrual health management for women's confidence and agency: a case study on the Thai/Burma border Wellington, New Zealand: Victoria University of Wellington; 2020. Available from: https://researcharchive.vuw.ac.nz/xmlui/handle/10063/9063.
15. Coker-Bolt P, Jansson A, Bigg S, Hammond E, Hudson H, Hunkler S, et al. Menstrual Education and Personal Hygiene Supplies to Empower Young Women in Haiti. OTJR (Thorofare N J). 2017;37(4):210-7. Epub 2017/07/25. doi: 10.1177/1539449217719866. PubMed PMID: 28737079.
16. Femme International. Monitoring & Evaluation Report Kilimanjaro Region 2017: Successes and lessons learned from the Thaweza program 2017. Available from: https://www.femmeinternational.org/wp-content/uploads/2018/09/Femme-International-ME-Report-2017.pdf.
17. Gade A, Hytti L. Menstrual health in Rhino Camp refugee settlement, West Nile, Uganda; pilot intervention report Kampala, Uganda and Copenhagen, Denmark: WoMena; 2017. Available from: https://www.susana.org/en/knowledge-hub/resources-and-publications/library/details/3506.
18. Geismar N. Participatory development and menstrual health management in South Africa: A case study of Project Dignity Brattleboro, Vermont, USA: School for International Training; 2018. Available from: https://digitalcollections.sit.edu/isp_collection/2846.
19. International Federation of Red Cross and Red Crescent Societies. Menstrual hygiene management (MHM) in emergencies: consolidated report 2016. Available from: https://watsanmissionassistant.org/wp-content/uploads/2018/10/menstrual-hygiene-management-in-emergencies_-consolidated-report-2016.pdf.
20. International Federation of Red Cross and Red Crescent Societies. Menstrual hygiene: what's the fuss? Piloting menstrual hygiene management (MHM) kits for emergencies in Bwagiriza refugee camp, Burundi 2013 [25 January 2021]. Available from: https://watsanmissionassistant.org/wp-content/uploads/2018/10/ifrc_mhm-kits-for-emergencies_case-study_final.pdf.
21. Kansiime C, Hytti L, Nalugya R, Nakuya K, Namirembe P, Nakalema S, et al. Menstrual health intervention and school attendance in Uganda (MENISCUS-2): a pilot intervention study. BMJ Open. 2020;10(2):e031182. Epub 2020/02/07. doi: 10.1136/bmjopen-2019-031182. PubMed PMID: 32024786.
22. Nalugya R, Tanton C, Hytti L, Kansiime C, Nakuya K, Namirembe P, et al. Assessing the effectiveness of a comprehensive menstrual health intervention program in Ugandan schools (MENISCUS): process evaluation of a pilot intervention study. Pilot Feasibility Stud. 2020;6:51. Epub 2020/04/30. doi: 10.1186/s40814-020-00585-2. PubMed PMID: 32346485.

23. Kuncio T. Pilot study findings on the provision of hygiene kits with reusable sanitary pads: United Nations High Commisioner for Refugees; 2018. Available from: https://data2.unhcr.org/en/documents/details/69059.
24. Mucherah W, Thomas K. Reducing barriers to primary school education for girls in rural Kenya: reusable pads' intervention. Int J Adolesc Med Health. 2017;31(3). Epub 2017/06/20. doi: 10.1515/ijamh-2017-0005. PubMed PMID: 28628478.
25. Murthy L. Green is the new colour for menstruation. Environmentally sustainable, Uger fabric pads, shows the way. Indian Institute of Science, Bangalore, India 2015 [27 July 2021]. Available from: http://jatansansthan.org/wp-content/uploads/2020/09/Icord-15-Grren-is-the-New-Colour-Of-Menstruation.pdf.
26. Hooper A, Loy E, Clayton F, Nabata K, Gao T, Shankar R, et al. Long-term feasibility, sustainability, and cost-benefit analysis of reusable menstrual hygiene management products in a population of adolescent females in a remote Himalayan community. J Investig Med. 2020;68 (Suppl 1):A176.
27. Nabata K, Clayton F. Comparing the long term feasibility of reusable menstrual hygiene management strategies in a population of adolescent females in a remote Himalayan community. J Investig Med. 2019;67:111-2.
28. Scott L, Montgomery P, Steinfield L, Dolan C, Dopson S. Sanitary pad acceptability and sustainability study 2013 [27 July 2021]. Available from: https://menstrualhygieneday.org/wp-content/uploads/2016/12/UniversityOxford_SanPads_Kenya_2013.pdf.
29. Amulen E. Utilization of modern re-usable menstrual pads among school going adolescent girls aged 10-19 years in Bidibidi refugee settlement, Yumbe District, Uganda Kampala, Uganda: Makerere University; 2019. Available from: http://makir.mak.ac.ug/handle/10570/7643.
30. Beksinska M, Nkosi P, Zulu B, Smit J. Acceptability of the menstrual cup among students in further education institutions in KwaZulu-Natal, South Africa. Eur J Contracept Reprod Health Care. 2020:1-6. Epub 2020/09/18. doi: 10.1080/13625187.2020.1815005. PubMed PMID: 32938248.
31. Borowski A. Are American women turning to reusable and greener menstrual products due to health and environmental pollution concerns? Rochester, NY, USA: Rochester Institute of Technology; 2011. Available from: http://scholarworks.rit.edu/theses/544/.
32. Crankshaw TL, Strauss M, Gumede B. Menstrual health management and schooling experience amongst female learners in Gauteng, South Africa: a mixed method study. Reprod Health. 2020;17(1):48. Epub 2020/04/16. doi: 10.1186/s12978-020-0896-1. PubMed PMID: 32293481.
33. Frank SF. Queering menstruation: Trans and non-binary identity and body politics. Sociol Inq. 2020;90(2):371-404.
34. Goodson CJ. The issue of blood - Urban Ministry in a rural setting: New Brunswick Theological Seminary; 2020. Available from: https://www.proquest.com/openview/83f43c198fca787826b4f94dde491032/1?pq-origsite=gscholar&cbl=18750&diss=y.
35. Hennegan J, Sol L. Confidence to manage menstruation at home and at school: findings from a cross-sectional survey of schoolgirls in rural Bangladesh. Cult Health Sex. 2020;22(2):146-65. Epub 2019/04/02. doi: 10.1080/13691058.2019.1580768. PubMed PMID: 30931818.
36. Kim HJ, Choi SY. Status of use of menstrual hygiene products and genital organ hygiene management in unmarried women. Korean J Women Health Nursing. 2018;24(3):265-75.
37. Sebert Kuhlmann A, Peters Bergquist E, Danjoint D, Wall LL. Unmet Menstrual Hygiene Needs Among Low-Income Women. Obstet Gynecol. 2019;133(2):238-44. Epub 2019/01/12. doi: 10.1097/aog.0000000000003060. PubMed PMID: 30633137.
38. Lamont JM, Wagner KM, Incorvati CG. The relationship of self-objectification and body shame to attitudes toward and willingness to use reusable menstrual products. Womens Reprod Health. 2019;6(1):1-16.
39. Lenia E. Assessment of knowledge, perceptions and practices of menstrual hygiene management among females aged 15-49 years in Bidibidi Refugee Settlement, Yumbe district, Uganda Kampala, Uganda: University of Makerere; 2019. Available from: http://makir.mak.ac.ug/handle/10570/7851.
40. Miiro G, Rutakumwa R, Nakiyingi-Miiro J, Nakuya K, Musoke S, Namakula J, et al. Menstrual health and school absenteeism among adolescent girls in Uganda (MENISCUS): a feasibility study. BMC Womens Health. 2018;18(1):4. Epub 2018/01/05. doi: 10.1186/s12905-017-0502-z. PubMed PMID: 29298699.
41. Moon G, Kim I, Kim H, Choe S, Jeon S, Cho J, et al. How can we improve knowledge and perceptions of menstruation? A mixed-methods research study. BMC Womens Health. 2020;20(1):214. Epub 2020/10/01. doi: 10.1186/s12905-020-01007-4. PubMed PMID: 32993611.
42. SNV. SNV Menstrual hygiene management baseline survey conducted in Lira, Mubende, Dokolo and Mukono districts Kampala, Uganda: SNV Uganda; 2014. Available from: https://snv.org/assets/explore/download/girls_in_control_baseline_report_-_uganda.pdf.
43. Tang KM, Li C, Kan C. Measurement of liquid transport properties of sanitary napkin with modified forced flow water transport tester. Fibers Polymers. 2019;20(12):2646-53.
44. Tang KM, Li C, Kan C. Instrumentation for measuring the wet frictional property of sanitary pads. Fibers Polymers. 2020;21(1):216-23.
45. Yilmaz SK, Bohara AK, Chowdhury SR. Touch for health: Use of Pavlovian processes with physical touch as a means to improve menstrual hygiene management initiatives, measured by willingness to pay. PharmacoEcon. 2020;4:263-76.
46. Sundberg F, Anderhell M. Washing and drying reusable sanitary pads Boras, Sweden: University of Boras; 2019. Available from: http://www.diva-portal.org/smash/record.jsf?pid=diva2%3A1368026&dswid=-1365.
47. Tiku SD. Design and development of feminine reusable pad without pad holder. Int J Clothing Science Technol. 2020;32(2):271-83. doi: 10.1108/IJCST-09-2018-0116.

48. Findlay PK. Radical innovation and taboo: How to encourage the acceptance of a new menstrual product in South Africa through behavioral design Johannesburg, South Africa: University of Johannesburg; 2020. Available from: http://hdl.handle.net/10210/415163.
49. Crofts T, Fisher J. Menstrual hygiene in Ugandan Schools: an investigation of low-cost sanitary pads. J Water Sanit Hyg Dev. 2012;2(1):50-8.
50. Gaybor J. Empowerment, destigmatization and sustainability: the co-construction of reusable menstrual technologies in the context of menstrual activism in Argentina. Gend Technol Dev. 2019;23(2):111-29. doi: 10.1080/09718524.2019.1643522.
51. Hennegan J, Dolan C, Steinfield L, Montgomery P. A qualitative understanding of the effects of reusable sanitary pads and puberty education: implications for future research and practice. Reprod Health. 2017;14(1):78. Epub 2017/06/29. doi: 10.1186/s12978-017-0339-9. PubMed PMID: 28655302.
52. Jewitt S, Ryley H. It's a girl thing: Menstruation, school attendance, spatial mobility and wider gender inequalities in Kenya. Geoforum. 2014;56:137-47.
53. Kambala C, Chinangwa A, Chipeta E, Torondel B, Morse T. Acceptability of menstrual products interventions for menstrual hygiene management among women and girls in Malawi. Reprod Health. 2020;17:185.
54. Ottsen PJ. Exploring girls' menstrual inclusion in India. Lund, Sweden: Lund University; 2020.
55. Schmitt ML, Clatworthy D, Ratnayake R, Klaesener-Metzner N, Roesch E, Wheeler E, et al. Understanding the menstrual hygiene management challenges facing displaced girls and women: findings from qualitative assessments in Myanmar and Lebanon. Confl Health. 2017;11:19. Epub 2017/10/20. doi: 10.1186/s13031-017-0121-1. PubMed PMID: 29046714.
56. Sommer M, Schmitt ML, Ogello T, Mathenge P, Mark M, Clatworthy D, et al. Pilot testing and evaluation of a toolkit for menstrual hygiene management in emergencies in three refugee camps in Northwest Tanzania. J Int Humanit Action. 2018. doi: 10.1186/S41018-018-0034-7.
57. VanLeeuwen C, Torondel B. Exploring menstrual practices and potential acceptability of reusable menstrual underwear among a Middle Eastern population living in a refugee setting. Int J Womens Health. 2018;10:349-60. Epub 2018/07/24. doi: 10.2147/IJWH.S152483. PubMed PMID: 30034256.
58. Vora S. The realities of period poverty: How homelessness shapes women's lived experiences of menstruation. In: Bobel C, Winkler IT, Fahs B, Hasson KA, Kissling EA, Roberts T, editors. The Palgrave Handbook of Critical Menstruation Studies. Singapore, Singapore: Springer Nature; 2020.
59. Natoli L. The last taboo: Research on menstrual hygiene management in the Pacific: Solomon Islands, Fiji, and Papua New Guinea 2017. Available from: https://healtheducationresources.unesco.org/library/documents/last-taboo-research-menstrual-hygiene-management-pacific-solomon-islands-fiji-andgay.
60. Kukkonen TM, Binik YM, Amsel R, Carrier S. An evaluation of the validity of thermography as a physiological measure of sexual arousal in a non-university adult sample. Arch Sex Behav. 2010;39:861-73.
61. EDANA. EDANA Guidelines for the testing of feminine hygiene products 2018 [24 January 2021]. Available from: https://www.edana.org/docs/default-source/international-standards/femcare-testing-guidelines-final.pdf.
62. Phillips-Howard PA, Nyothach E, Ter Kuile FO, Omoto J, Wang D, Zeh C, et al. Menstrual cups and sanitary pads to reduce school attrition, and sexually transmitted and reproductive tract infections: a cluster randomised controlled feasibility study in rural Western Kenya. BMJ Open. 2016;6(11):e013229. Epub 2016/11/25. doi: 10.1136/bmjopen-2016-013229. PubMed PMID: 27881530.
63. RealRelief. Safepad 2020 [24 January 2021]. Available from: https://realreliefway.myshopify.com/products/safepad.
64. Garikipati S. Asymmetric information in menstrual health and implications for sustainability: Insights from India Liverpool, UK: University of Liverpool; 2020. Available from: https://ideas.repec.org/p/liv/livedp/202015.html.
