# Supplementary material for: Exploring menstrual products: A systematic review and meta-analysis of reusable menstrual pads for public health internationally
Source: PLoS One. 2021 Sep 24;16(9):e0257610. doi: 10.1371/journal.pone.0257610 (PMC8462722; doi:10.1371/journal.pone.0257610)
Supplement: Attachment — Submitted filename: PONE_response_04aug21.docx [file pone.0257610.s004.docx]

Journal requirements:
1. Please ensure that your manuscript meets PLOS ONE's style requirements, including those for file naming. The PLOS ONE style templates can be found at
<https://journals.plos.org/plosone/s/file?id=wjVg/PLOSOne_formatting_sample_main_body.pdf> and
<https://journals.plos.org/plosone/s/file?id=ba62/PLOSOne_formatting_sample_title_authors_affiliations.pdf>

*Response: The manuscript has been reformatted following the guidelines of the journal.*

2. Please include the tables within the main manuscript.

*Response: The tables have been included within the main manuscript.*

3. Thank you for stating the following in the Funding Section of your manuscript:
“Joint Global Health Trials Initiative (UK-Medical Research Council/Department for International Development/Wellcome Trust/Department of Health and Social Care, grant MR/N006046/1)**”**

We note that you have provided funding information that is not currently declared in your Funding Statement. However, funding information should not appear in the Acknowledgments section or other areas of your manuscript. We will only publish funding information present in the Funding Statement section of the online submission form.
Please remove any funding-related text from the manuscript and let us know how you would like to update your Funding Statement. Currently, your Funding Statement reads as follows:
“Joint Global Health Trials Initiative (UK-Medical Research Council/Department for International Development/Wellcome Trust/Department of Health and Social Care, grant MR/N006046/1)**”**

*Response: We removed the funding-section at the end of the text of the manuscript, which read as follows:*

*“****Funding***

*This study is funded by the Joint Global Health Trials Initiative (UK-Medical Research Council/Department for International Development/Wellcome Trust/Department of Health and Social Care, grant MR/N006046/1). The funders had no role in the design of the study, the collection, analysis, and interpretation of data, or in writing the manuscript. The corresponding author had full access to all data in the study and had final responsibility to submit for publication.”*
Please include your amended statements within your cover letter; we will change the online submission form on your behalf.

*Response: It would be nice if the section below could be added to that statement; however, we do not think this essential if that would be a problem.*

*“The funders had no role in the design of the study, the collection, analysis, and interpretation of data, or in writing the manuscript. The corresponding author had full access to all data in the study and had final responsibility to submit for publication.”*

4) Please review your reference list to ensure that it is complete and correct. If you have cited papers that have been retracted, please include the rationale for doing so in the manuscript text, or remove these references and replace them with relevant current references. Any changes to the reference list should be mentioned in the rebuttal letter that accompanies your revised manuscript. If you need to cite a retracted article, indicate the article’s retracted status in the References list and also include a citation and full reference for the retraction notice.

*Response: We have reviewed the reference list for completeness, following the Plos One guidelines and using the Plos One Endnote template. We removed Wilson et al (2019), because this manuscript could not be retrieved in a published or accepted version. The text, figures and tables were updated accordingly.*

Reviewers' comments:

Review Comments to the Author

5) Reviewer #1: A highlighted research area needs to public health was reviewed by the authors. Need some experimental data for supporting this review paper. The authors collected more numbers of research papers and consisted as a informative review.

- Outstanding review work was done by the authors. A lot of assortment date related to exploring menstrual products.
- These review paper, authors done a systemic review related to reusable menstrual pads.
- The topic is very highlighted and useful for public health, the authors collected and reviewed quantitative and qualitative studies that reported on leakage, acceptability, or safety of RMPs.

*Response: We are pleased that the reviewer was positive about the manuscript, and we thank the reviewer for the time spent on this. The reviewer request for experimental data made no sense in the context of this systematic review and meta-analysis, where data pertaining to the study aims and objectives were fully extracted, analysed, and interpreted. As such, we have subsequently not made any changes based on these comments.*

6) Reviewer #2: The manuscript entitled "Exploring menstrual products: a systematic review and meta-analysis of reusable menstrual pads for public health internationally" by Anna Maria van Eijk et al., is very well-written and summarizes the current status of the reusable menstrual products (RMPs). Given the demand for the eco-friendly and biodegradable alternatives, menstrual pads and diapers compose a huge portion of dumped thrash, posing serious hazard to the environment. An awareness about the availability of reusable alternatives to the single-use menstrual pads is need of the hour. This review summarizes the use, and limitations of the RMPs, incorporating statistical analysis of the demographically diverse women and transgenders. Such reviews will be of great use in highlighting the improvements needed for the future RMP products and I recommend publication of this article as is

*Response: We thank the reviewer for the positive comments and have not made any changes based on these comments.*

7) While revising your submission, please upload your figure files to the Preflight Analysis and Conversion Engine (PACE) digital diagnostic tool, <https://pacev2.apexcovantage.com/>. PACE helps ensure that figures meet PLOS requirements. To use PACE, you must first register as a user. Registration is free. Then, login and navigate to the UPLOAD tab, where you will find detailed instructions on how to use the tool. If you encounter any issues or have any questions when using PACE, please email PLOS at [figures@plos.org](mailto:figures@plos.org). Please note that Supporting Information files do not need this step.

*Response: The figures have been uploaded as requested. Note that Figure 1 (the flow chart) was adapted to reflect the exclusion of the Wilson 2019 reference.*
