# Supplementary material for: Exploring menstrual products: A systematic review and meta-analysis of reusable menstrual pads for public health internationally
Source: PLoS One. 2021 Sep 24;16(9):e0257610. doi: 10.1371/journal.pone.0257610 (PMC8462722; doi:10.1371/journal.pone.0257610)
Supplement: Attachment — Submitted filename: REVIEWER COMMENTS.doc [file pone.0257610.s003.doc]

- Outstanding review work was done by the authors. A lot of assortment date related to exploring menstrual products.
- These review paper, authors done a systemic review related to reusable menstrual pads.
- The topic is very highlighted and useful for public health, the authors collected and reviewed quantitative and qualitative studies that reported on leakage, acceptability, or safety of RMPs.
